# Structure and mechanism of CutA, RNA nucleotidyl transferase with an unusual preference for cytosine

**DOI:** 10.1093/nar/gkaa647

**Published:** 2020-08-12

**Authors:** Deepshikha Malik, Kamil Kobyłecki, Paweł Krawczyk, Jarosław Poznański, Aleksandra Jakielaszek, Agnieszka Napiórkowska, Andrzej Dziembowski, Rafał Tomecki, Marcin Nowotny

**Affiliations:** Laboratory of Protein Structure, International Institute of Molecular and Cell Biology, Trojdena 4, Warsaw 02-109, Poland; Laboratory of RNA Biology and Functional Genomics, Institute of Biochemistry and Biophysics, Polish Academy of Sciences, Pawinskiego 5a, Warsaw 02-106, Poland; Laboratory of RNA Biology, International Institute of Molecular and Cell Biology, Trojdena 4, Warsaw 02-109, Poland; Institute of Biochemistry and Biophysics, Polish Academy of Sciences, Pawinskiego 5a, Warsaw 02-106, Poland; Laboratory of Protein Structure, International Institute of Molecular and Cell Biology, Trojdena 4, Warsaw 02-109, Poland; Structural Biology Center, International Institute of Molecular and Cell Biology, Trojdena 4, Warsaw 02-109, Poland; Laboratory of RNA Biology, International Institute of Molecular and Cell Biology, Trojdena 4, Warsaw 02-109, Poland; Institute of Genetics and Biotechnology, Faculty of Biology, University of Warsaw, Pawinskiego 5a, Warsaw 02-106, Poland; Laboratory of RNA Biology and Functional Genomics, Institute of Biochemistry and Biophysics, Polish Academy of Sciences, Pawinskiego 5a, Warsaw 02-106, Poland; Institute of Genetics and Biotechnology, Faculty of Biology, University of Warsaw, Pawinskiego 5a, Warsaw 02-106, Poland; Laboratory of Protein Structure, International Institute of Molecular and Cell Biology, Trojdena 4, Warsaw 02-109, Poland

## Abstract

Template-independent terminal ribonucleotide transferases (TENTs) catalyze the addition of nucleotide monophosphates to the 3′-end of RNA molecules regulating their fate. TENTs include poly(U) polymerases (PUPs) with a subgroup of 3′ CUCU-tagging enzymes, such as CutA in *Aspergillus nidulans*. CutA preferentially incorporates cytosines, processively polymerizes only adenosines and does not incorporate or extend guanosines. The basis of this peculiar specificity remains to be established. Here, we describe crystal structures of the catalytic core of CutA in complex with an incoming non-hydrolyzable CTP analog and an RNA with three adenosines, along with biochemical characterization of the enzyme. The binding of GTP or a primer with terminal guanosine is predicted to induce clashes between 2-NH_2_ of the guanine and protein, which would explain why CutA is unable to use these ligands as substrates. Processive adenosine polymerization likely results from the preferential binding of a primer ending with at least two adenosines. Intriguingly, we found that the affinities of CutA for the CTP and UTP are very similar and the structures did not reveal any apparent elements for specific NTP binding. Thus, the properties of CutA likely result from an interplay between several factors, which may include a conformational dynamic process of NTP recognition.

## INTRODUCTION

Template-independent terminal ribonucleotide transferases (TENTs) (1) catalyze the addition of nucleotide monophosphates to the 3′-end of various RNA molecules and such additions play an important role in determining fate of these RNAs (2). The importance of the addition of A tails has been long recognized—canonical poly(A) polymerases polyadenylate nearly all eukaryotic mRNAs and the poly(A) tail bound by poly(A) binding proteins is essential for mRNA stability and efficient translation (3,4). The first and rate-limiting step in mRNA decay is shortening of the poly(A) tail, followed by further degradation (5).

A growing number of so-called poly(U) polymerases (PUPs), also termed terminal uridyl transferases (TUTases), which mostly add uridyl ribonucleotides to the 3′-end of RNA, have recently been described. These are involved in multiple pathways of RNA regulation but usually induce the accelerated decay of target RNA molecules (6,7) through a mechanism in which uridine tagging at the 3′-end of a transcript by a TUTase is followed by decapping and 5′-3′ or 3′-5′ exonucleolytic degradation (8–10). In addition to 3′ uridine tagging, 3′ guanylation and cytidylation in different species have also been reported (11–13). Moreover, addition of a short, up to 7 nt long, heteropolymeric C/U-rich tail (3′ CUCU-tagging) has been observed in filamentous fungi, such as *Aspergillus nidulans* (14,15). Addition of such C/U extensions to the poly(A) tail of mRNA leads to rapid decay (14). Tails with atypical composition are likely added to mRNAs in other organisms (16,17), but little is known about the mechanistic and structural basis of these unusual enzymatic activities.

TENTs belong to the DNA polymerase β superfamily. Their catalytic domains contain a palm domain (also termed catalytic NTase domain) and fingers domain (also termed PAP/OAS SBD) (1). The basis of differences in their activity originates from the nucleotide recognition motif (NRM) that is located in the central domain and RNA binding domain, which may be located at the N- or C-terminus or inserted into the catalytic domain (2). The NRM loop harbors the determinants of differences between canonical and non-canonical TENTs. However, the precise molecular mechanisms that underlie NTP and primer preference are not fully understood.

Humans have three uridyl transferases (TENT1/TUT1, TENT3A/ZCCHC11/TUT4 and TENT3B/TUT7/ZCCHC6) and in *S. pombe*, the main non-canonical uridyl transferase is Cid1 (6,7). Cid1 can add both uridines and adenosines *in vitro* but is specific for U *in vivo* (18–21). Cid1 has been extensively characterized structurally (22–26). The reported structures of Cid1 revealed an element that is responsible for UTP recognition as the incoming nucleotide, which comprises the key histidine residue (His336). Its mutation to Ala or Asn converted Cid1 from processive poly(U) polymerase to a processive poly(A) polymerase (23,25). Uracil selection by His336 was postulated to involve flipping of this histidine residue (22). Thorough structural characterization has also been published for human TUTases (27,28), trypanosomal NTases (29,30), as well as for yeast and mammalian poly(A) polymerases (for example see: (31–33)).

C/U modification in *A. nidulans* involves the addition of short tails that consist of cytidines and uridines by a Cid1 homologue, termed CutA (15). This modification is added to poly(A) tails to alleviate the need for their shortening prior to decapping (15). *In vitro* analysis of the activity of CutA from *A. nidulans* and its homologue from thermophilic fungus, *Thielavia terrestris*, was recently performed (34). Site-directed mutagenesis and 3′-rapid amplification of cDNA ends (3′-RACE) analysis coupled with high-throughput sequencing showed that CutA prefers CTP as the incoming nucleotide over other nucleotides, even in the presence of excess adenosine triphosphate (ATP). However, CutA was not processive for C, as it could add only two cytidines to the 3′-end. CutA was also shown to be processive for adenosines (34). Finally, the protein was unable to incorporate guanosines. The structural basis for these biochemical properties of CutA was unknown. Therefore, the present study sought to solve its crystal structures in complex with various substrates to elucidate its mechanism of action. Here, we report structures of the catalytic core of *T. terrestris* CutA (TtCutA) in the apo form and its complexes with a non-hydrolyzable CTP analog and A_3_ RNA. We also biochemically characterize its activity. Based on these structures, we propose elements of the protein that are responsible for preference for certain incoming NTPs and particularly the inability of CutA to use guanosine triphosphate (GTP). We also describe elements that are responsible for primer binding and the role they may play in selection against RNAs that terminate with guanosine and catalysis of the processive polymerization of adenosines. Comparisons with other TENTs show that the mechanisms that are responsible for NTP and RNA primer selection are complex and likely involve not only preferential binding but also more dynamic molecular recognition mechanisms.

## MATERIALS AND METHODS

### Bacterial strains

The following *Escherichia coli* strains were used in this study: MH1 (*E. coli araD lacX74 galU hsdR hsdM rpsL*) and BL21-CodonPlus(DE3)-RIL (Agilent; *E. coli* B F^–^*ompT hsdS*[r_B_^–^ m_B_^–^] *dcm*^+^ Tet^r^*gal λ[DE3] endA* Hte [*argU ileY leuW* Cam^r^]).

### Oligonucleotides, plasmids and cloning

The plasmids and oligonucleotides used in this study are listed in Supplementary Tables S1 and 2, respectively. All restriction enzymes and Phusion DNA polymerase for the amplification of inserts were from Thermo Fisher Scientific. DNA purification kits (DNA Plasmid Mini and Gel-Out) were from A&A Biotechnology.

The pCC19-pCC32 plasmids used for the expression of truncated TtCutA variants were generated by sequence- and ligation-independent cloning (SLIC) (35) in *E. coli* MH1 strain. Inserts were amplified using respective primers listed in Supplementary Table S2 and were cloned into BamHI-XhoI sites of the pET28M N-6xHis-SUMOTag vector.

The pCC33, pCC34, pCC35, pCC36 and pCC37 plasmids for the expression of TtCutA^240–603^ truncation variants with single amino acid substitutions were generated by site-directed mutagenesis with oligonucleotide pairs: mutN397Af-mutN397Ar, mutA400Gf-mutA400Gr, mutN403Af-mutN403Ar, mutR557Af-mutR557Ar and mutR557Hf-mutR557Hr, respectively, using pCC25 as the template. The presence of introduced mutations was verified by sequencing the inserts with appropriate primers, listed in Supplementary Table S2.

### Heterologous expression of proteins in *E. coli*

For the expression of TtCutA variants, the *E. coli* BL21-CodonPlus(DE3)-RIL strain was transformed with appropriate plasmids (pCC19-pCC37). Transformants were cultivated at 18°C for 48 h in 1 l or 50 ml (for large- and small-scale purification, respectively) of Auto Induction Medium (AIM) Super Broth Base including Trace elements (Formedium), which contained 2% glycerol, 50 μg/ml kanamycin and 34 μg/ml chloramphenicol. Larger cultures were inoculated from a pre-culture that was grown in standard Luria-Broth (LB) medium that contained both antibiotics.

### Small-scale purification of truncated TtCutA variants

A bacterial pellet from 50 ml of liquid culture was resuspended in 5 ml of lysis buffer (50 mM Tris–HCl pH 7.4, 50 mM NaCl, 10 mM imidazole, 10 mM 2-mercaptoethanol, 1 mM phenylmethylsulfonyl fluoride [PMSF]) and subjected to 60 cycles of sonication (30 s of full-power sonication, followed by a 30 s pause) in a Bioruptor XL device (Diagenode). The homogenate was centrifuged at 10 000 × *g* for 15 min at 4°C. The supernatant was then loaded onto a Protino 96 Ni-IDA plate (Macherey-Nagel) that was pre-equilibrated with lysis buffer. Following protein binding, the resin was sequentially washed with 2 ml of low-salt (LS) buffer (50 mM Tris-HCl pH 7.4, 50 mM NaCl, 10 mM imidazole), 1 ml of high-salt (HS) buffer (50 mM Tris–HCl pH 7.4, 1 M NaCl, 10 mM imidazole) and 2 ml of LS buffer. Bound proteins were recovered by three consecutive rounds of elution with 0.25 ml of buffer E (50 mM Tris–HCl pH 7.4, 50 mM NaCl, 300 mM imidazole), which were then pooled. SUMO cleavage was performed using 10 μg of the homemade SUMO protease in buffer E. Purified proteins were analyzed by standard sodium dodecyl sulfate-polyacrylamide gel electrophoresis (SDS-PAGE).

### Large-scale protein purification of truncated TtCutA variants

Following the centrifugation of 1 l of liquid culture at 4500 rpm in a Sorvall H6000A/HBB6 swinging-bucket rotor for 15 min at 4°C, the bacterial pellet was resuspended in 70 ml of lysis buffer, incubated with lysozyme (50 μg/ml; Roth) for 30 min in a cold room and then broken in an EmulsiFlex-C3 High Pressure homogenizer at 1500 Bar. The homogenate was centrifuged in a Sorvall WX Ultra Series ultracentrifuge (F37L rotor) at 32 000 rpm for 45 min at 4°C. The extract was then used for protein purification using the ÄKTA Xpress system (GE Healthcare), employing nickel affinity chromatography on an ÄKTA-compatible 5 ml column that was filled with Ni-NTA Superflow resin (Qiagen). The column was equilibrated with 25 ml of LS buffer before extract loading. After protein binding, the resin was sequentially washed with 35–60 ml of LS buffer, 25 ml of HS buffer and 20 ml of LS buffer. SUMO protease cleavage was performed for 8 h by on-column digestion with 100 μg of the homemade enzyme that was resuspended in 5 ml of buffer E, followed by buffer exchange to LS on the desalting column and final separation of the desired protein from the cutout 6xHis-SUMO epitope and SUMO protease on the Ni-NTA column. Size-exclusion chromatography was then performed using a Hiload 16/60 Superdex S200 column (GE Healthcare) by applying 5 ml of the eluate from the affinity chromatography step using 1.2 column volumes of gel-filtration (GF) buffer (20 mM Tris–HCl pH 7.4, 50 mM NaCl). Purified proteins were analyzed by standard SDS-PAGE.

### Purification of TtCutA^240-603^ for crystallization

The bacterial pellet was lysed by sonication in buffer L (50 mM Tris–HCl pH 7.5, 2 M NaCl, 40 mM imidazole, 5% [v/v] glycerol, 5 mM 2-mercaptoethanol). To clarify the resulting lysate, it was spun down at 40 000 rpm (Beckmann coulter, 45Ti rotor) and the supernatant was loaded on a HisTrap column (GE Healthcare) that was pre-equilibrated with buffer A (50 mM Tris–HCl pH 7.5, 0.5 M NaCl, 40 mM imidazole, 5% [v/v] glycerol, 5 mM 2-mercaptoethanol). The column was then washed first with buffer A and then with the same buffer with higher imidazole concentration (75 mM). The protein was then eluted with buffer A that contained 300 mM imidazole. To remove the 6×His-SUMO tag, pooled fractions of the eluate were incubated with homemade SUMO protease. Overnight dialysis was then performed against buffer D (20 mM Tris–HCl pH 7.5, 0.5 M NaCl, 20 mM imidazole, 5% [v/v] glycerol, 5 mM 2-mercaptoethanol). The dialyzed sample was then reapplied on the HisTrap column, and the flow-through that contained the protein of interest was concentrated using an Amicon Centrifugal Filter Device (Millipore). The concentrated protein was applied on a Hiload 16/60 Superdex S200 size exclusion column that was equilibrated with buffer B (20 mM Tris–HCl pH 8.0, 100 mM NaCl, 0.5 mM Tris[2-carboxyethyl]phosphine [TCEP], 0.5 mM ethylenediaminetetraacetic acid [EDTA]) for crystallization. The eluted protein was then concentrated again. For long-term protein storage at −80°C, the gel filtration column was equilibrated in buffer B that contained 10% glycerol. The same purification procedure was used for purification of the selenomethionine-substituted protein.

### Crystallography

The crystallization trials for TtCutA^240–603^ were performed using the sitting-drop vapor diffusion method at 18°C. The protein buffer contained 20 mM HEPES-KOH pH 7.5, 100 mM NaCl, 5 mM TCEP and 0.5 mM EDTA. The protein concentration was 12.5 mg/ml. For apo CutA^240–603^, the initial crystallization conditions were obtained from an Index screen (Hampton Research). The crystals of selenomethionine-substituted protein were obtained in 0.1 M HEPES-KOH pH 7.5, 0.2 M lithium sulfate monohydrate and 25% polyethylene glycol 3350. These crystals diffracted X-rays to 2.2 Å resolution. The dataset was collected at PETRA III, EMBL Hamburg, at a selenium peak wavelength of 0.9785 Å. The crystals belonged to the *C* 2 space group and contained two molecules per asymmetric unit. The apo CutA^240–603^ structure was solved using a data set collected from a selenomethionine-substituted protein using single-wavelength anomalous diffraction in the Autosol module of Phenix (36). The model was further built manually in COOT (37) with the help of a homology model based on the TENT3B/TUT7/ZCCHC6 structure (Protein Data Bank [PDB] ID: 5W0B) and generated using SWISS-MODEL (38). The final model was refined against a data set collected using native protein crystals, which produced better model statistics than the SeMet data (Table 1). This dataset was collected at beamline 14.1 at Berliner Elektronenspeicherring-Gesellschaft für Synchrotronstrahlung (BESSY) (39) at a wavelength of 0.9760 Å.

**Table 1.** Data collection and refinement statistics

	apo CutA (SeMet)	apo CutA (native)	CutA-CMPCPP	CutA-A_3_
**Data collection**				
Space group	*C* 2	*C* 2	*C* 2 2 2_1_	*P* 1
Cell dimensions				
*a*, *b*, *c* (Å)	90.6, 71.1, 145.9	90.0, 70.2, 145.3	88.2, 93.5, 86.8	56.9, 56.9, 149.8
α, β, γ (°)	90, 104.1, 90	90, 103.2, 90	90, 90, 90	79.8, 87.1, 75.8
Resolution (Å)*	50–2.19 (2.32–2.19)	46.6-2.25 (2.39–2.25)	32.0–1.45 (1.54–1.45)	33.2–1.90 (2.01–1.90)
CC_1/2_*^,^**	99.6 (57.1)	99.6 (55.6)	99.9 (64.9)	99.7 (58.9)
*I* / σI*	8.07 (1.15)	8.43 (1.31)	13.0 (1.43)	8.49 (1.20)
Completeness (%)*	99.6 (97.7)	98.3 (97.9)	99.6 (98.5)	96.1 (94.3)
Redundancy*	6.8 (6.6)	3.5 (3.5)	12.9 (12.2)	3.6 (3.6)
**Refinement**				
Resolution (Å)		46.6–2.25	32.0–1.45	33.2–1.90
No. of unique reflections***		41 363 (1641)	63 467 (3172)	136 374 (6817)
*R*_work_/*R*_free_		19.6/24.1	15.6/18.1	17.8/21.3
No. atoms		5880	6197	12 529
Protein		5531	5778	11 264
Nucleic acid			43	252
Ion/Water		349	376	1013
*B*-factors (Å^2^)		51.1	24.6	41.1
Protein		50.9	23.3	39.5
Nucleic acid			17.9	91.1
Ion/Water		53.3	35.4	45.9
RMSD				
Bond length (Å)		0.002	0.012	0.009
Bond angle (°)		0.480	1.206	0.945

The data collection statistics are based on a single crystals for each structure.

*Values in parentheses are for highest-resolution shell.

**CC1/2—correlation coefficient between the average intensities in two parts of the unmerged data, each with a random half of the measurements of each unique reflection.

***Number of reflections used for *R*_free_ calculation in parentheses.

Crystals of CutA^240–603^ with CMPCPP (Cytidine-5′-[(α,β)-methyleno]triphosphate, a non-hydrolyzable CTP analog) in the presence of 5 mM CaCl_2_ were obtained by co-crystallization of the 0.78 mM ligand with protein in 0.1 M sodium formate and 20% polyethylene glycol 3350. X-ray diffraction data were collected at PETRA III Hamburg. The crystals belonged to the *C* 2 2 2_1_ space group, contained one molecule per asymmetric unit, and diffracted X-rays to 1.4 Å resolution. The structure was solved by molecular replacement using the Phaser module (40) in Phenix. The apo CutA^240–603^ structure was used as a search model.

Crystals of CutA^240–603^ plus A_3_ RNA were obtained by soaking apo CutA^240–603^ crystals for 3–24 h with 2.5 mM A_3_ RNA in the presence of 5 mM MgCl_2_ in a buffer that contained 0.1 M Bis-Tris pH 6.5, 0.2 M lithium sulfate monohydrate and 25% polyethylene glycol 3350. The crystals diffracted to 1.9 Å resolution and belonged to the *P* 1 space group. The X-ray diffraction data were collected at PETRA III Hamburg. The structure was solved by molecular replacement using Phaser and the apo CutA^240–603^ structure as the search model. The asymmetric unit contained four molecules of the complex.

All of the datasets were processed using XDSAPP GUI (41). The structures were refined in phenix.refine (36) with manual building in Coot (37). Final refinement statistics are presented in Table 1. The apo structure had one Ramachandran plot outlier and the other two had none. The percentages of residues that were located in the most favorable region of the Ramachandran plot, according to Molprobity (42), were the following: apo CutA—98.1%, CutA-CMPCPP—98.6% and CutA-A_3_—97.9%. Simulated annealing composite omit maps were calculated using Phenix (36). Structural figures were generated using Pymol (PyMOL Molecular Graphics System, version 2.2, Schrödinger, LLC; http://pymol.org/).

### Nucleotidyl transferase assays

The PAGE-purified RNA oligonucleotides with fluorescein amidite (FAM) modification at the 5′-end were purchased from Future Synthesis (Poznan, Poland; http://futuresynthesis.com/). *In vitro* nucleotidyl transferase reactions were performed in a 20 μl volume using buffer that contained 10 mM Tris–HCl pH 8.0, 50 mM NaCl, 1 mM MnCl_2_, 1 mM DTT and either individual NTP (1 mM) or mixtures of four different NTPs (either 0.25 mM each or 0.77 mM ATP, 0.077 mM GTP, 0.077 mM CTP and 0.077 mM UTP). Unless otherwise indicated, 1 μM of the appropriate TtCutA variant and 1 μM of 5′ FAM-labeled oligoribonucleotide substrate was used. The reactions were performed at 37°C and stopped at various time points by collecting an aliquot of the reaction mixture and adding it to an equal volume of formamide loading dye (95% formamide, 0.025% SDS, 20 mM EDTA, 0.03% bromophenol blue, 0.03% xylene cyanol in 1× Tris-borate-EDTA [TBE]), followed by flash freezing in liquid nitrogen. Following thermal denaturation at 90°C for 5 min, reaction products were resolved either in denaturing 20% polyacrylamide/8 M urea/1× TBE gels or in 10% sequencing PAGE if single-nucleotide resolution was required. A Typhoon FLA 9000 laser scanner (GE Healthcare) was used to visualize fluorescently labeled RNA molecules.

### NTP affinity determination by fluorescence anisotropy measurements

The assay was performed in 96-well, black, flat-bottom polystyrene NBS plates (Corning 3650) in a total reaction volume of 50 μl. The substrate was used at a concentration of 50 nM, and the protein concentration ranged from 5 to 3000 nM to generate a saturation curve. The binding of CutA to ATP-Cy5 (Jena Bioscience; https://www.jenabioscience.com/) was tested in the presence of Ca^2+^ ions. The binding assay was performed in the presence of 10 mM Tris–HCl pH 8.0, 2.5 mM CaCl_2_, 100 mM NaCl and 0.5 mM DTT at 25°C. The reactions were performed in triplicate. The reaction mixture was shaken for 5 s and anisotropy was measured using a Tecan Infinite M1000 microplate reader at an excitation wavelength of 635 nm and emission wavelength of 670 nm with a bandwidth of 5 nm. The saturation curve was fitted using one site-specific binding and GraphPad Prism software.

### Fluorescence anisotropy competition assays to measure binding affinity of unlabeled NTPs

The competition assays were carried out in order to measure the affinity of unlabeled NTPs for CutA. The assays were carried out in the same 96-well Corning 3650 plates as mentioned above. The labeled ligand used for these experiments was ATP-Cy5 at a concentration of 50 nM, and protein concentration was 250 nM. The concentration of unlabeled NTP competitor varied from 50 nM to 500 μM. Competition assays for all four NTPs were carried out in triplicates. The anisotropy measurements were performed as described in the previous section. For each NTP the saturation curves were analyzed globally in Origin 2019 using the competitive binding model:

$$\begin{equation*}{{\rm{A}}_{{\rm{{\rm obs}}}}}\left( {\left[ {NTP} \right]} \right)\ = {A_{free}}\ + \frac{{{A_{{\rm bound}}} - {A_{{\rm free}}}}}{{1 + \left[ {NTP} \right]/I{C_{50}}}}\end{equation*}$$

where IC_50_ was fitted globally, while *A*_free_ and *A*_bound_ that describe anisotropy of free ATP-Cy5 and apparent anisotropy of 50 nM ATP-Cy5 in the presence of 250 nM CutA were optimized individually for each experiment.

### 3′-RACE sequencing

Nucleotide compositions of 3′-extensions that were added by different TtCutA^240–603^ variants were analyzed using 3′-RACE and high-throughput sequencing. The sequences of the oligonucleotides that were used in the 3′-RACE-seq procedure are listed in Supplementary Table S2.

#### RNA purification

After RNA visualization, gel fragments with reaction products were excised, frozen in liquid nitrogen and shredded with a pipette tip. RNA was recovered from acrylamide by elution with 350 μl of the gel elution buffer (100 mM Tris–HCl pH 8.0, 150 mM NaCl, 12.5 mM EDTA, 1% SDS) in the presence of 800 μl of phenol:chloroform (1:1, v:v) on a rotating wheel at room temperature for 16 h. After centrifugation at 13 500 × *g* for 15 min at room temperature, the aqueous phase was transferred to a new tube and RNA was precipitated with isopropanol in the presence of 1 μl of glycogen (20 mg/ml) at −20°C for 16 h. RNA was pelleted by centrifugation at 21 000 × *g* for 30 min at 4°C and washed twice with 80% ethanol. The RNA pellet was then suspended in 11 μl of RNase-free water.

#### 3′-end adapter ligation

Purified RNA (10.7 μl) was mixed with 2 μl of T4 RNA Ligase Reaction buffer (New England Biolabs), 1 μl of 30 μM RA3 adapter oligonucleotide, 4.8 μl of 50% PEG 8000 (New England Biolabs) and 0.5 μl of RiboLock RNase inhibitor (40 U/μl; Thermo Fisher Scientific). After 2 min of incubation at 70°C, the tube was placed on ice and 1 μl of T4 RNA Ligase 2, Truncated (200 U/μl; New England Biolabs) was added. The reaction was performed for 1 h at 25°C.

#### Reverse transcription

RNA with an adapter that was ligated to its 3′-end was used as a template for reverse transcription. RTP primer (1.5 μl of 20 μM) was annealed by heating the mixture at 75°C for 5 min, followed by incubation at 37°C for 30 min and then at 25°C for 15 min. A 10 μl aliquot of the reaction was transferred to a new tube, mixed with 4 μl of 5× First Strand Buffer (Invitrogen), 1 μl of 10 mM dNTP Mix, 0.5 μl of RiboLock RNase Inhibitor, 2 μl of 100 mM DTT and 1.5 μl of RNase-free H_2_O and incubated at 42°C for 2 min. Afterward, 1 μl of SuperScript III Reverse Transcriptase (Invitrogen) was added, and reverse transcription was performed at 42°C for 1 h. Enzyme inactivation was performed by heating the reaction mixture at 70°C for 30 min.

#### Polymerase chain reaction amplification

Two-step amplification was performed for high-throughput RNA-seq of the 3′-RACE products. The first-round polymerase chain reaction (PCR) reaction mixture contained 20 μl of first-strand cDNA, 0.2 mM dNTP Mix, 20 μM of the RA5_ss22-RPI_X primer pair (with Illumina index for RNA-seq), 5× Phusion HF Buffer, Phusion DNA polymerase and sterile water to a volume of 40 μl. The PCR conditions were the following: initial denaturation at 98°C for 30 s, 25 cycles of amplification (denaturation at 98°C for 10 s, annealing at 55°C for 10 s and extension at 72°C for 30 s) and final extension at 72°C for 5 min. The second-round PCR reaction mixture consisted of 1 μl of the first-round PCR products, 0.2 mM dNTP Mix, 20 μM of the RP1-RPI_X primer pair, 5× Phusion HF Buffer, Phusion DNA polymerase and sterile water to a volume of 50 μl. The PCR profile that is described above was essentially the same as in the first round, but with only five cycles of amplification. The PCR products were analyzed by electrophoresis in 2% agarose gels.

#### Purification of 3′-RACE libraries

The PCR products were further purified using Agencourt AMPure XP magnetic beads (Beckman Coulter). Briefly, 48 μl of the PCR products was mixed with 48 μl of the beads, incubated for 15 min at room temperature and then placed on a magnetic stand. After 5 min, the supernatant was discarded, and the beads were washed twice with 100 μl of 80% ethanol and air-dried for 7 min. Elution was performed by suspending the beads in 20 μl of sterile water, followed by 5 min of incubation at room temperature. The magnetic stand was used to separate purified DNA from the beads.

#### High-throughput sequencing of 3′-RACE products

3′-RACE-seq library concentrations were measured using the KAPA Library Quantification Universal Kit (Kapa Biosystems). Sequencing was performed using an Illumina MiSeq System and MiSeq Reagent Kit (version 3, 2 × 75 bp read length) by applying standard paired-end run procedures.

#### Bioinformatic data analysis of 3′-RACE-seq results

Only reads that contained sequences of RNA substrates in the sense orientation were used. Sequences that contained an entirely correct 22 nt stretch of the respective RNA substrate (ss22-A_4_ or ss22-U_4_) at the 5′-end and an entirely correct sequence of the RA3 oligonucleotide (21 nt) at the 3′-end were first selected using cutadapt 1.18. All of the remaining sequences and sequences that contained an insert longer than 100 nt or only a short stretch of ACTG were discarded as contaminants. A summary of reads that passed the filters is presented in Supplementary Table S3.

All of the remaining analyses were conducted using in-house R scripts, with functions that were contained in the Biostrings Bioconductor package. Briefly, the sequences were first reversed to have the ultimate 3′-end at the first position. Substrate-encoded nucleotides (up to four adenosines or uridines were then removed with TrimLRPatterns (from the Biostrings package). Nucleotide frequencies at each position were calculated using the consensus Matrix function (Biostrings). Plots were prepared using the ggplot2 package.

## RESULTS AND DISCUSSION

### CutA adopts a canonical fold of poly(A)/(U) polymerase

The primary aim of the present study was to solve crystal structures of CutA to understand the molecular basis of its unique biochemical properties, including (i) processive RNA polymerization exclusively for adenosines, (ii) a complete lack of activity for GTP as the incoming nucleotide, (iii) an inability to extend primers that end with guanosines and (iv) a preference for incoming cytidines over uridines in the nucleotide transfer reaction.

We first attempted to crystallize the recombinant *T. terrestris* CutA truncated variant that was utilized in previous studies (TtCutA^216–617^; TtCutA_tr1) (34), and was largely devoid of unstructured N- and C-terminal parts of the protein but encompassed the entire fingers (PAP/OAS SBD) and palm (catalytic NTase) domains (Supplementary Figure S1A). These trials did not result in any crystals. Therefore, based on *in silico* predictions, we designed over a dozen protein variants that were truncated up to predicted helices at both N- and C-termini (Supplementary Figure S1A and B) and tested their expression and purification efficiency in a soluble and intact form (Supplementary Figure S1C–E). This approach eventually led to a TtCutA^240–603^ fragment that contained the catalytic core of the protein. We next performed extensive crystallization trials for this protein. For simplicity, this construct is hereinafter referred to as CutA unless otherwise specified.

We first obtained crystals of the apo form of the protein, the structure of which was solved by SeMet SAD and refined against a data set from native protein crystal to 2.25 Å resolution and an *R*_free_ of 24.1%. We next performed extensive co-crystallization and crystal soaking experiments with non-hydrolyzable analogues of nucleotide triphosphates and various RNA oligonucleotides, which resulted in two crystal forms and structures. We first obtained crystals of CutA in complex with a non-hydrolyzable CTP analogue (CMPCPP). The structure was solved by molecular replacement using the apo structure and refined to 1.45 Å resolution with an *R*_free_ of 18.1%. The other crystal was CutA in complex with an A_3_ RNA oligonucleotide, which was obtained by soaking crystals of apo protein crystals with RNA. This structure was solved to 1.9 Å resolution and refined to an *R*_free_ of 21.3%. Diffraction data and refinement statistics are shown in Table 1. Sample electron density maps are shown in Supplementary Figure S2.

**Figure 1.**
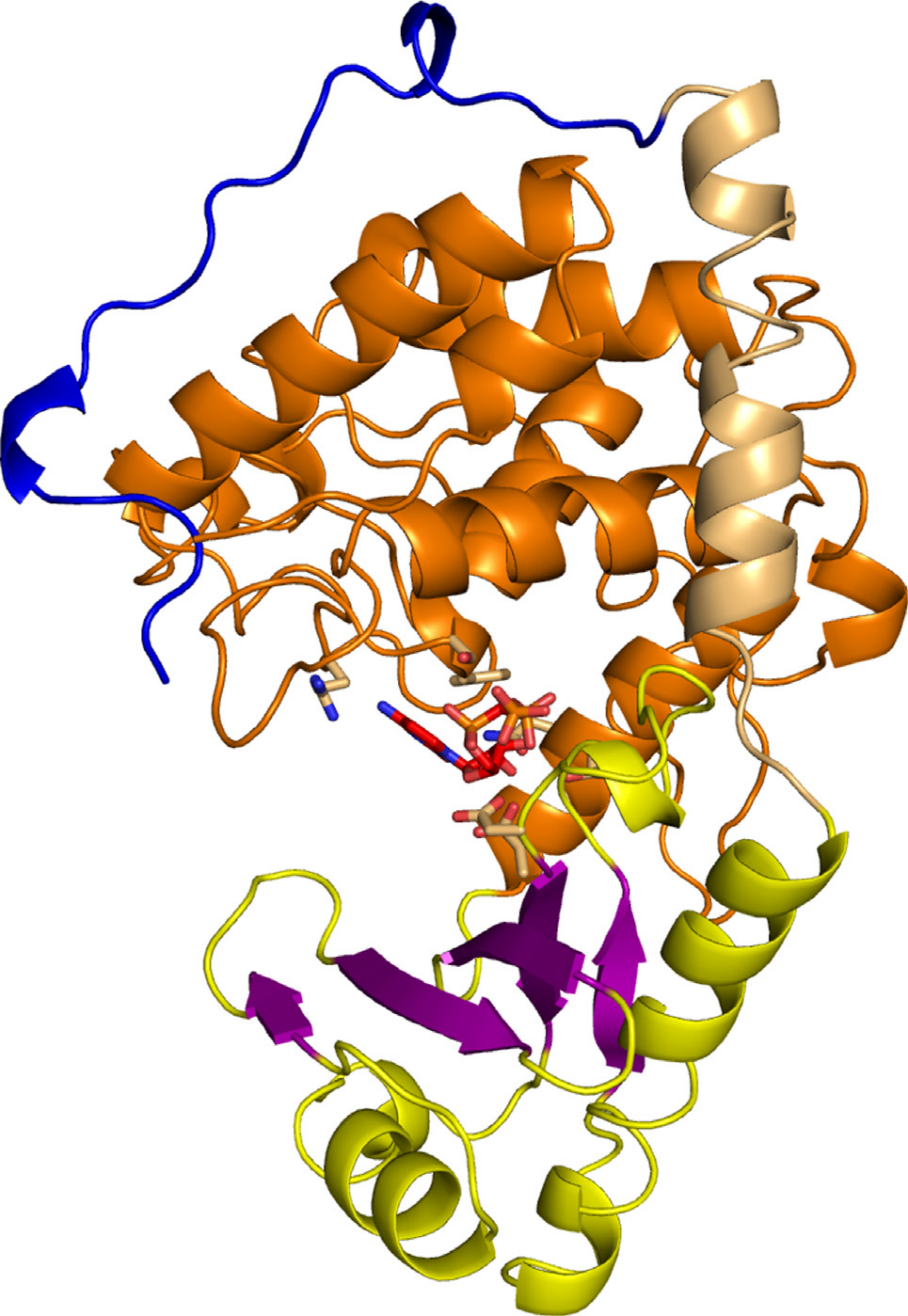
Overall structure and subdomains of the catalytic core of CutA (CutA–CMPCPP complex). The palm domain is shown in yellow, with β-strands in purple and finger domain in orange. The N-terminal α-helix is shown in a lighter shade of orange. The N-terminal loop is shown in blue. The incoming CMPCPP is shown as red sticks and active site residues are shown as orange sticks.

**Figure 2.**
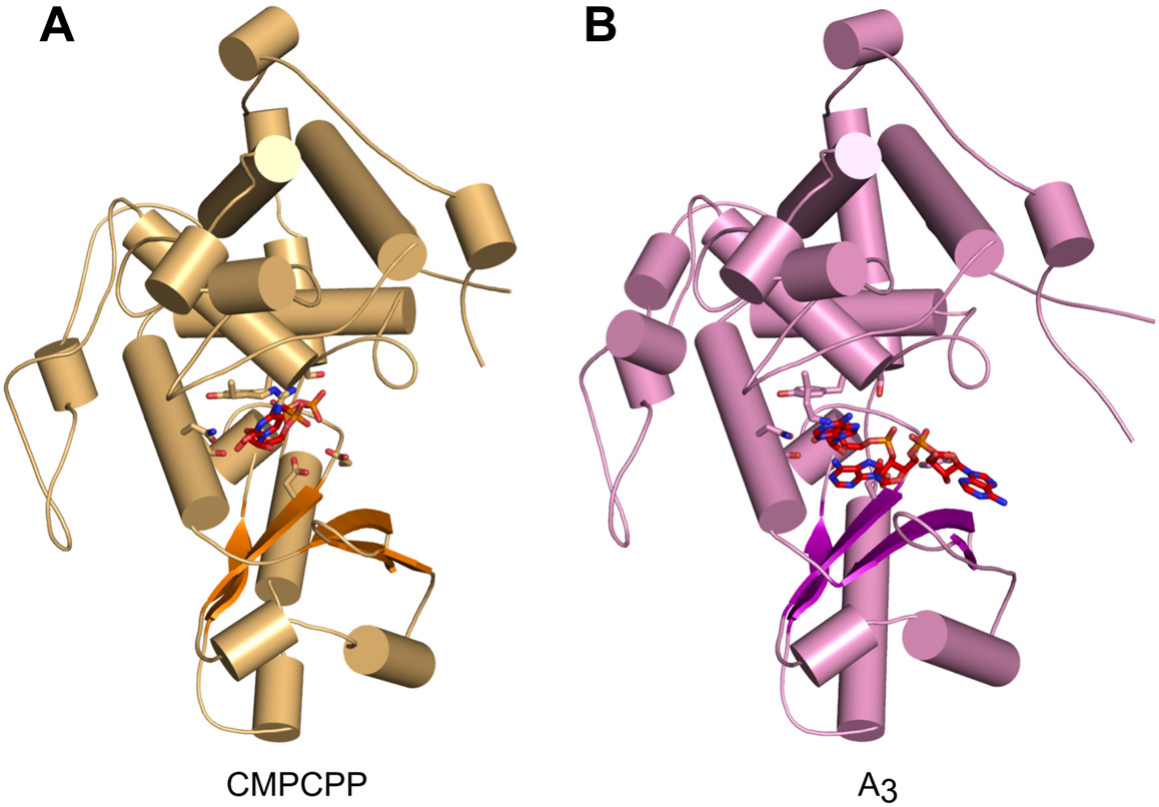
Overall structures of CutA complexes. (**A**) Structure of CutA–CMPCPP complex. The protein is shown as an orange cartoon, with β-strands in a darker shade of orange. Incoming nucleotide is shown as red sticks and active site residues are shown as orange sticks. (**B**) Structure of CutA–A_3_ complex. The protein is shown as pink cartoon, with β-strands in purple. The RNA is shown as red sticks and active site residues are shown as pink sticks.

As expected, the overall protein structure is very similar to other TENTs and adopts a bilobal architecture (Figure 1). The polymerase palm domain comprises a five-stranded β-sheet and two helices and harbors active-site carboxylates (see below). The finger subdomain is located within the C-terminal portion of the catalytic core and is composed of six α-helices. An N-terminal helix of the catalytic core interacts with one side of the finger subdomain, likely stabilizing arrangement of the two subdomains (Figure 1). The active site and incoming nucleotide are located between the palm and finger subdomains (Figure 2). The protein conformation in the apo state and in both complexes with nucleic acid is essentially the same (Figure 2 and Supplementary Figure S3). We observed small movements of the palm and finger domains relative to each other. Such movements have been proposed to be important elements of the catalytic cycle of a related polymerase, Cid1 (22).

The structure of CutA is very similar to its closest structural homologues, Cid1 and TENT3B/TUT7/ZCCHC6. For example, the proteins from the CutA-CMPCPP complex and Cid1-UTP complex (PDB ID: 4FH5) (25) could be superimposed with a root mean square deviation (RMSD) of 1.9 Å (215 C-α atoms). Similarly, CutA could be superimposed on TENT3B from the TENT3B-U_5_ complex (PDB ID: 5W0M) (28) with an RMSD of 1.7 Å (234 C-α atoms). Compared with Cid1 and TENT3B, the unique feature of CutA is an N-terminal region of the catalytic core, which forms a loop that extends the N-terminal α-helix. The loop wraps around the finger subdomain and points toward the palm subdomain (Figure 1). This loop very likely plays a role in stabilizing the overall structure of the enzyme. Another element that differs in the structures of PUPs is a loop at the back of the catalytic core (residues 460–498 in CutA) (25), which adopts a different structure in CutA than in closest homologues.

### The structures reveal features discriminating against GTP

**Figure 3.**
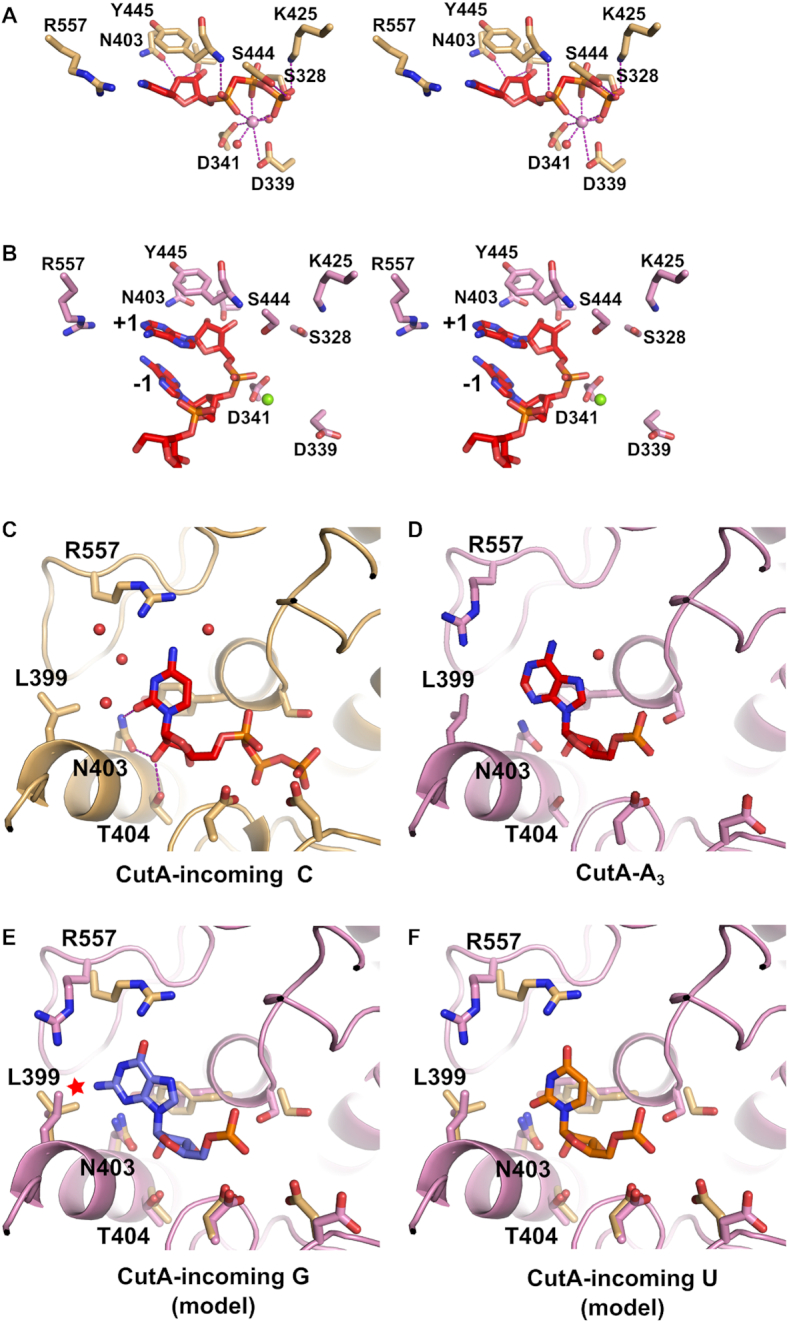
The active site of CutA. (**A**) Active site in the CutA–CMPCPP complex (stereoview). The incoming nucleotide is shown as red sticks and active site residues are shown as orange sticks. The Ca^2+^ ion is shown as a pink sphere. Purple dashed lines show metal ion coordination and contacts between the nucleotide and the protein. (**B**) Active site in the CutA–A_3_ complex (stereoview). RNA is shown as red sticks and active site residues are shown as pink sticks. The Mg^2+^ ion is shown as a green sphere. (**C**) Binding of the base of the incoming nucleotide in CutA–CMPCPP complex. Purple dashed lines show selected contacts. (**D**) Binding of the base of nt +1 in the CutA–A_3_ complex. (**E**) Model of binding of the incoming guanosine triphosphate. Potential clash is indicated with a red star. (**F**) Model of binding of the incoming uridine triphosphate. In (E) and (F), protein models for both CutA-A_3_ and CutA-CMPCPP structures are shown (colored as in [A] and [B]) to visualize different possible conformations of the active site.

The active site of CutA adopts a configuration that is very similar to related NTases (Figure 3A and B; Supplementary Figure S4). The core of the active site is formed by two carboxylates (Asp339 and Asp341) that would coordinate two catalytic divalent metal ions in a fully formed active center. In the CutA-CMPCPP structure, one Ca^2+^ ion was observed, coordinated by side chains of both aspartates, and non-bridging oxygens of all three phosphates of the incoming nucleotide. It corresponds to metal ion B in the canonical two-metal ion mechanism of polymerases. Two water molecules complete the coordination sphere. Based on the homologous structures, the second metal ion should be coordinated by a non-bridging oxygen of the α-phosphate group of the incoming nucleotide. However, it was not visible in our electron density maps and most likely absent because of the chemical modification of CMPCPP.

The contacts that stabilize the incoming nucleotide in the CutA-CMPCPP structure include binding of the γ phosphate by Lys425, Ser328 and Ser444 (Figure 3A). A hydrogen bond forms between the backbone amide group of Tyr445 and non-bridging oxygen of the α phosphate. This phosphate and negative charge that builds on it in the transition state are also likely stabilized by the positive pole of the helical dipole of the α-helix, which begins at Tyr445. The side chain of Tyr445 is located between the ribose ring and base of the incoming nucleotide, stabilizing it through van der Waals interactions. The 2′-OH group of the nucleotide forms a network of hydrogen bonds with Asn403 and Thr404 side chains (Figure 3C), thus ensuring preference for incoming ribonucleotides over deoxyribonucleotides.

In the CutA–A_3_ complex structure, the 3′-terminal nucleotide of the RNA occupies the position of the incoming nucleotide (Figure 3B). Therefore, it represents a product complex that forms after incorporation of the nucleotide but before translocation of the RNA to allow binding of the next incoming nucleotide. In this structure, a single Mg^2+^ ion is observed that is located closer to the position of the divalent metal ion A from the canonical fully formed polymerase active site. This metal ion interacts with Asp341 and the phosphate group between the last and penultimate nucleotide of the RNA.

The structures that are reported herein provide insights into binding of the base of the incoming cytidine and adenosine phosphate (nt +1 in the CutA–A_3_ complex; Figure 3C and D). For cytidine, a few contacts were observed between the base and the protein. The strongest contact may be the hydrogen bond between the O2 carbonyl of the base and side chain of Asn403. As noted above, this residue also forms a hydrogen bond with the 2′-OH group of the incoming nucleotide, thus playing a dual role in recognition of the base and ribonucleotide versus deoxyribonucleotide selection. Moreover, Arg557 is located 3.3 Å from 4-NH_2_ of the cytosine, potentially forming a weak polar interaction. In the CutA–A_3_ structure, Asn403 also forms a hydrogen bond with N3 of the base at position +1. The side chain of Arg557 is positioned 3.3 Å from N6 amine and N1 imine nitrogen atoms of the +1 adenine base and it adopts a different conformation than in CutA–CMPCPP complex. As a consequence of a different Arg557 conformation, the nearby Glu551 also adopts different rotamers in the two structures. Two alternative conformations were modeled for this residue in the CutA–CMPCPP complex.

To obtain insights into discrimination against the incoming GTP, we substituted the base of nt +1 with guanine in the structural model. This substitution showed that the 2-NH_2_ group of guanine would clash with Leu399 (Figure 3E), which would explain why CutA cannot use GTP as a substrate. We also changed the nt +1 in our structural model to uracil (Figure 3F). However, we did not readily identify any difference in base contacts that would explain the preference for CTP over UTP as the incoming nucleotide. The only strong contact between the protein and base of the NTP was formed between a side chain of Asn403 and O2 carbonyl of the base; this would be the same for both CTP and UTP. Factors other than simple preferential binding, possibly involving dynamic conformational states, likely underlie discrimination between CTP and UTP.

Cid1 can incorporate uridine processively and has no activity with CTP as the incoming nucleotide (25). The active sites of Cid1 and CutA are nearly identical and the pockets binding the base of the incoming nucleotide are very similar (Supplementary Figure S4). The main difference is the nature of one of the key residues involved in base recognition (Arg557 in CutA versus His336 in Cid1). In crystal structures of Cid1, similar to our structures and predictions for CutA, contacts with the base of the incoming NTP are essentially the same for uridine and cytidine (25). It has been suggested that a single water molecule in Cid1 that interacts with the imino group of uracil may participate in this differential recognition. However, a single water molecule-mediated contact would be unlikely to lead to a profound difference between the efficient processive polymerization of uridines and no activity for cytidine, as observed for Cid1. Therefore, similar to CutA, the preference of Cid1 for uridine over cytidine cannot be fully explained by contacts that are observed in the crystal structures.

### CutA possesses specific elements for primer binding

**Figure 4.**
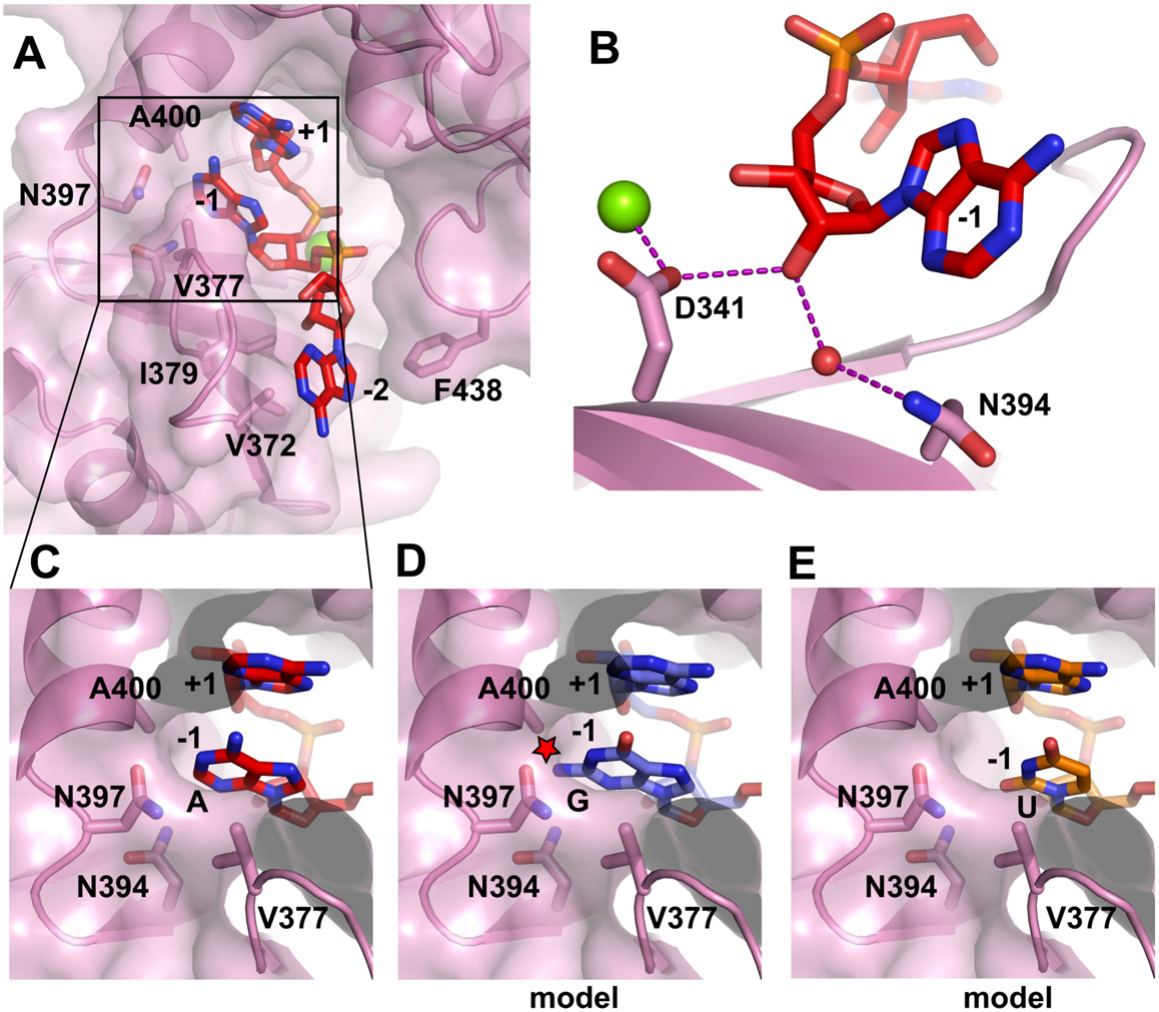
Binding of the primer in CutA–A_3_ complex. (**A**) The protein is shown in transparent surface representation and secondary structures are shown as a cartoon. Side chains that are involved in primer binding are shown as sticks and labeled. The RNA is shown as red sticks. (**B**) Close-up view of interactions between 2′-OH of nt −1 and the protein. (**C**) Interactions of the base of nt −1. (**D**) Modeling of guanine in position nt −1. Potential clashes are indicated with a red star. (**E**) Modeling of uracil in position nt −1.

The CutA–A_3_ complex structure reveals the mode of binding of the RNA substrate (Figure 4A). All three nucleotides of the RNA are visible in the density maps (Supplementary Figure S2B). As mentioned above, the first nucleotide from the 3′-end (nt +1) occupies the position of the incoming nucleotide, thus indicating that this structure represents the product complex. The bases of nt +1 and nt −1 are engaged in typical base stacking interactions. The 2′-OH group of nt −1 forms a hydrogen bond with Asp341, which also participates in coordination of the catalytic metal ion at the active site (Figure 4B). Thus, sensing 2′-OH at the 3′-terminus of the primer is coupled with assembly of the active site, ensuring that only RNA is extended. This mechanism is shared with related TENTs, such as Cid1 (24) and Tailor (43,44). Another 2′-OH interaction occurs with a water molecule, which in turn forms a weak hydrogen bond with a side chain of Asn394 (Figure 4B). These interactions likely serve as additional specificity determinants for RNA at the end of the primer. The base of nt −1 is located in a tight pocket that is partially formed by a side chain of Ala400 (Figure 4C). The base edge (imine nitrogen N1) is hydrogen-bonded with a side chain of Asn397. The exposed 5′-side surface of the base forms van der Waals interactions with the side chain of Val377 (Figure 4A). We call the element that is formed by this side chain a ‘shelf’.

We replaced adenine with guanine at nt −1 in our structural model (Figure 4D). This modeling exercise implied that the 2-NH_2_ group would clash with side chains of Asn394, Asn397 and Ala400. The last of these residues is located in an α-helix, so it is unlikely to undergo a conformational change to alleviate the clashes. When we replaced adenine with uracil in our structural model, we found that smaller bases would form much less efficient van der Waals interactions with the side chain of Val377 (i.e. the ‘shelf’; Figure 4E). Moreover, the smaller pyrimidine base could not reach sufficiently far to form a hydrogen bond with Asn397.

These observations may explain the preference of CutA for primers ending with adenosine. The interactions between the base of adenosine and the protein (hydrogen bond with Asn397 and interactions with the ‘shelf’) should be stronger than for bases of pyrimidine nucleotides (C or U). In Cid1, TENT1/TUT1 TUTase, TENT3B/TUT7/ZCCHC6 and Tailor Asn397 is conserved; in TENT3A/ZCCHC11/TUT4 it is replaced with arginine, which should be able to reach further and form contacts with a pyrimidine. However, in one of the TENT3B/TUT7/ZCCHC6 structures (PDB ID: 5W0N) (28), Asn1124 (the equivalent of Asn397 from CutA) forms a hydrogen bond with the O2 carbonyl group of uracil. Therefore, the involvement of Asn397 and its equivalents in nucleobase binding may be more complex.

**Figure 5.**
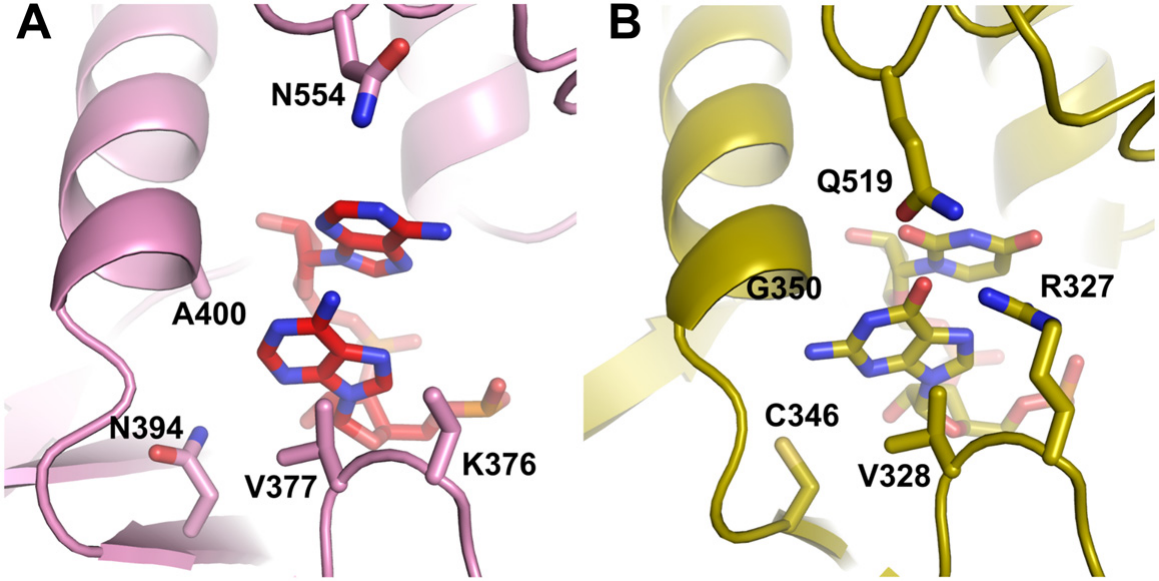
Comparison of nt −1 binding by CutA (**A**) and Tailor (**B**). Secondary structures are shown as a cartoon. Side chains that are involved in primer binding are shown as sticks and labeled.

Tailor is an interesting member of the poly(U) polymerase family. It possesses a UTP recognition motif with a conserved histidine (His522) and it specifically recognizes and uridylates 3′-terminal guanosines (45,46). This is a key difference between Tailor and CutA, since the latter has no activity on RNAs that terminate with guanosine (see below). The determinants of 3′-terminal guanine base specificity in Tailor are interactions that are formed by the guanidine group of Arg327 (Figure 5). Its equivalent in CutA is Lys376, which in the CutA–A_3_ structure is located in the vicinity of the phosphate group between nt −1 and nt −2, but it does not form any interactions with the RNA. Another Tailor residue that is involved in the recognition of nt -1 is Gln519, which forms water-mediated interactions with its base. The CutA equivalent is Asn554, which does not interact with the RNA in our structure, either directly or through water molecules. Thus, guanine-binding residues are not present in CutA. Perhaps even more important differences between CutA and Tailor can be found in the pocket that binds the base of nt −1. CutA residue Ala400, which we predicted would form a clash with the N2 amine group of the guanine base, is replaced with glycine (Gly350) in Tailor (Figure 5). Moreover, another CutA residue whose side chain was predicted to form a clash with the 2-NH_2_ of guanine (Asn394) is replaced in Tailor with a smaller Cys345. Both changes make the base-binding pocket in Tailor larger, potentially allowing accommodation of the 2-NH_2_ of guanine (Figure 5). The trajectories of the N-termini of helices that harbor Ala400 and Gly350 and preceding loops are different in the two proteins, likely because the loop is one amino acid shorter in Tailor than in CutA. This difference may also contribute to the preference for the 3′-terminal guanine base. The length of this loop and presence of Ala versus Gly in the helix may be good predictors of the ability of PUPs to recognize 3′-terminal guanosines.

**Figure 6.**
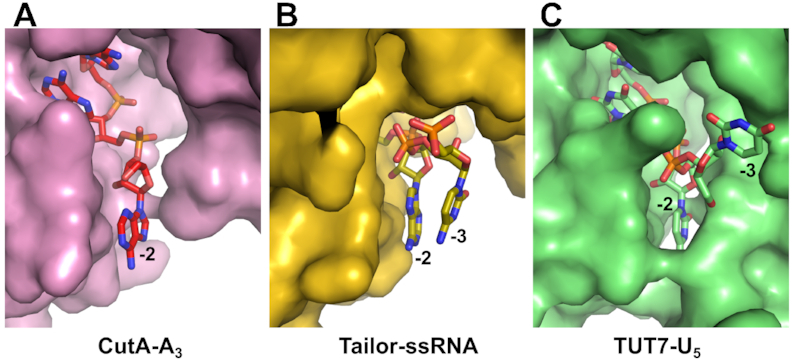
Binding of the nt −2 of primer by CutA (**A**), Tailor (**B**) and TENT3B/TUT7/ZCCHC6 (**C**). The proteins are shown in surface representation and RNA as sticks. Structures used: PDB ID: 6I0V (Tailor) and PDB ID: 5W0M (TENT3B/TUT7/ZCCHC6).

The third nucleotide of the RNA in the CutA-A_3_ complex (nt −2) is located in a narrow cleft between the finger and palm subdomains (Figure 4A). It forms multiple van der Waals interactions with side chains of Val372, Ala375, Phe438 and Ala437. We predict that these interactions would be more efficient for purines that have a larger surface of aromatic rings, which would further contribute to CutA processivity during poly(A) polymerization. The examples of other structures in which nt −2 was visualized are TENT3B/TUT7/ZCCHC6 with U_5_ (PDB ID: 5W0M) and dsRNA (PDB ID: 5W0O) (28) and Tailor with AGU (PDB ID: 5Z4A), AGUU (PDB ID: 5Z4D) U_6_ (PDB ID: 6I0U) and mixed sequence RNA (PDB IDs: 6I0V) (43,44). In all of these structures, the conformation and position of nt −2 are very similar and the interactions with the 3′ surface of the base, that involve regions around Val372 in CutA, are conserved (Figure 6). However, in each of the three enzymes the other flank of the cleft is very different (Figure 6). In Tailor, this other surface is further away from RNA, and the 5′ surface of the base of nt −2 stacks with the base of nt −3 (Figure 6B). In TENT3B/TUT7/ZCCHC6, the cleft is very narrow. One of its sides is formed by a unique mobile zinc-knuckle domain, and it completely and specifically buries uridine (Figure 6C) (28). Therefore, the structure of the cleft that binds nt −2 is another important determinant of the specificity of TENTs for the primer sequence.

In summary, our structures reveal elements that are responsible for primer binding. The base of nt −1 interacts with Asn397 and Val377 (‘shelf’), and the base of nt −2 is located in a protein cleft. Our modeling exercise also identified CutA residues that exclude the 2-NH_2_ group of guanine in position nt −1. Equivalent residues in Tailor are different, thus explaining the dramatically different activity of these two enzymes on RNA primers that end with guanosine.

### Crystallized CutA variant recapitulates properties of the full-length protein

To gain further insights into the mechanism of action of CutA, we performed a series of biochemical experiments using RNA primers with 22 nt of the random sequence (termed ss22; see Supplementary Table S2 for the sequence) and 4-nt long stretches of adenosines, cytidines, guanosines or uridines at the 3′ terminus. Unless otherwise specified, we used in the biochemical experiments the same protein fragment that was crystallized (CutA^240–603^).

**Figure 7.**
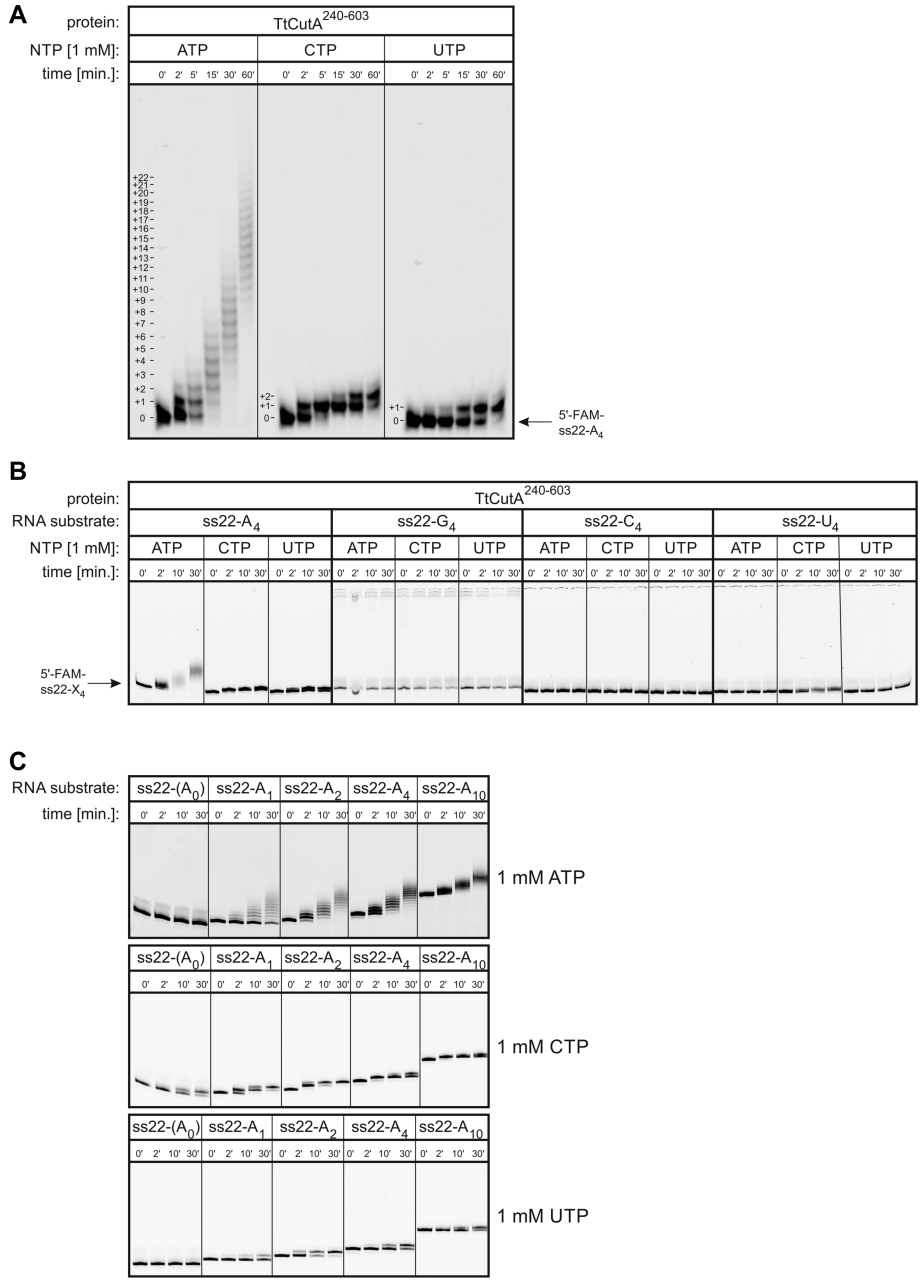
Biochemical properties of CutA^240–603^. (**A**) Nucleotidyl transferase activity assays were performed using ss22-A_4_ RNA oligonucleotide as a substrate (position indicated with an arrow). Reactions with NTPs (indicated on top of the gel) were terminated after the time points indicated above the lanes. The number of nucleotides that were added is indicated on the left of the reaction products. (**B**) Dependence of activity on the sequence at the 3′-end of the RNA primer. Assays were performed as in (A) but using ss22-X_4_ oligonucleotides that ended with 4-nt-long stretches of adenosines, guanosines, cytidines or uridines as indicated above the lanes. The position of RNA substrates is marked with an arrow. (**C**) Effect of the length of the adenosine stretch at the 3′-end of the ss22 RNA substrate on activity with different NTPs. Assays were performed as in (A) but using ss22-A_n_ oligonucleotides with no adenosine or a different number of adenosines at their 3′-ends as indicated above the lanes.

To check whether the catalytic activity of CutA^240–603^ recapitulates properties of the full-length protein, an assay was first performed using the ss22-A_4_ primer and ATP, CTP or UTP as an NTP substrate. Similar to full-length CutA, the truncated variant exhibited high processivity with ATP, while it added predominantly two cytidines or one uridine under the same conditions (Figure 7A).

Next, we investigated how nucleotide composition of the RNA primer 3′-end impacts CutA^240–603^ polymerization activity in the presence of ATP, CTP or UTP. We excluded GTP from the analysis because we knew from previous studies that the recombinant enzyme under limited protein concentration conditions is unable to utilize it as a substrate (34). The primer that ended with adenosines was extended in the presence of any tested NTP, albeit with variable processivity. The only other extended RNA substrate was the one that terminated with uridines, which was elongated exclusively when CTP was used as an NTP substrate (Figure 7B). Oligoribonucleotides with four consecutive guanosines or cytidines at the 3′-end were not extended under the same conditions, regardless of which NTP was present in the reaction. Considering these results and our previous data (34), only primers with 3′-terminal adenosines or uridines were tested in the subsequent experiments.

To further analyze the way in which the 3′-end of the RNA substrate affects the efficiency of polymerization by CutA^240–603^, assays in the presence of ATP, CTP or UTP were performed for a series of primers that contained a variable number of 3′-terminal adenosines. Notably, primers without adenosines at the 3′-end were not extended with ATP (Figure 7C). The addition of a single adenosine significantly increased polymerization efficacy, but full processivity was achieved for oligoribonucleotides that contained at least two adenosines (Figure 7C). These observations were consistent with the structural data. As described above, in the CutA-A_3_ structure, 2 nt of the primer were visualized, and both form interactions with the protein. Base of nt −1 forms interactions with the ‘shelf’ element and Asn397 and base of nt −2 is located in a narrow protein cleft (Figure 4). Intriguingly, in contrast to ATP, when CTP was used as the incoming nucleotide, the substrate that lacked pre-added adenosines was extended by 1 nt, but at least two 3′-terminal adenosines were required to enable extension with two cytidines (Figure 7C). In contrast, not even a single uridine was incorporated into the RNA primer that was devoid of terminal adenosines. The reaction in the presence of UTP occurred when at least one adenosine was present at the 3′-end of the primer, but the reaction was more efficient when two terminal adenosines were present (Figure 7C). Notably, stretches of 4 or 10 terminal adenosines slightly inhibited reaction efficiencies for both CTP and UTP compared with the ss22−A_2_ RNA primer (Figure 7C). These quite complex interdependencies are difficult to explain based on the structures that are presented herein and imply a complex interplay between the nature of the incoming nucleotide and the 3′-terminal nucleotide of the primer. A better explanation of this interplay would require further studies, including detailed molecular dynamics simulations. Nevertheless, the crystallized truncated variant of CutA generally recapitulates the enzymatic properties of the full-length protein.

### CutA binds GTP with lower affinity compared with other NTPs

**Figure 8.**
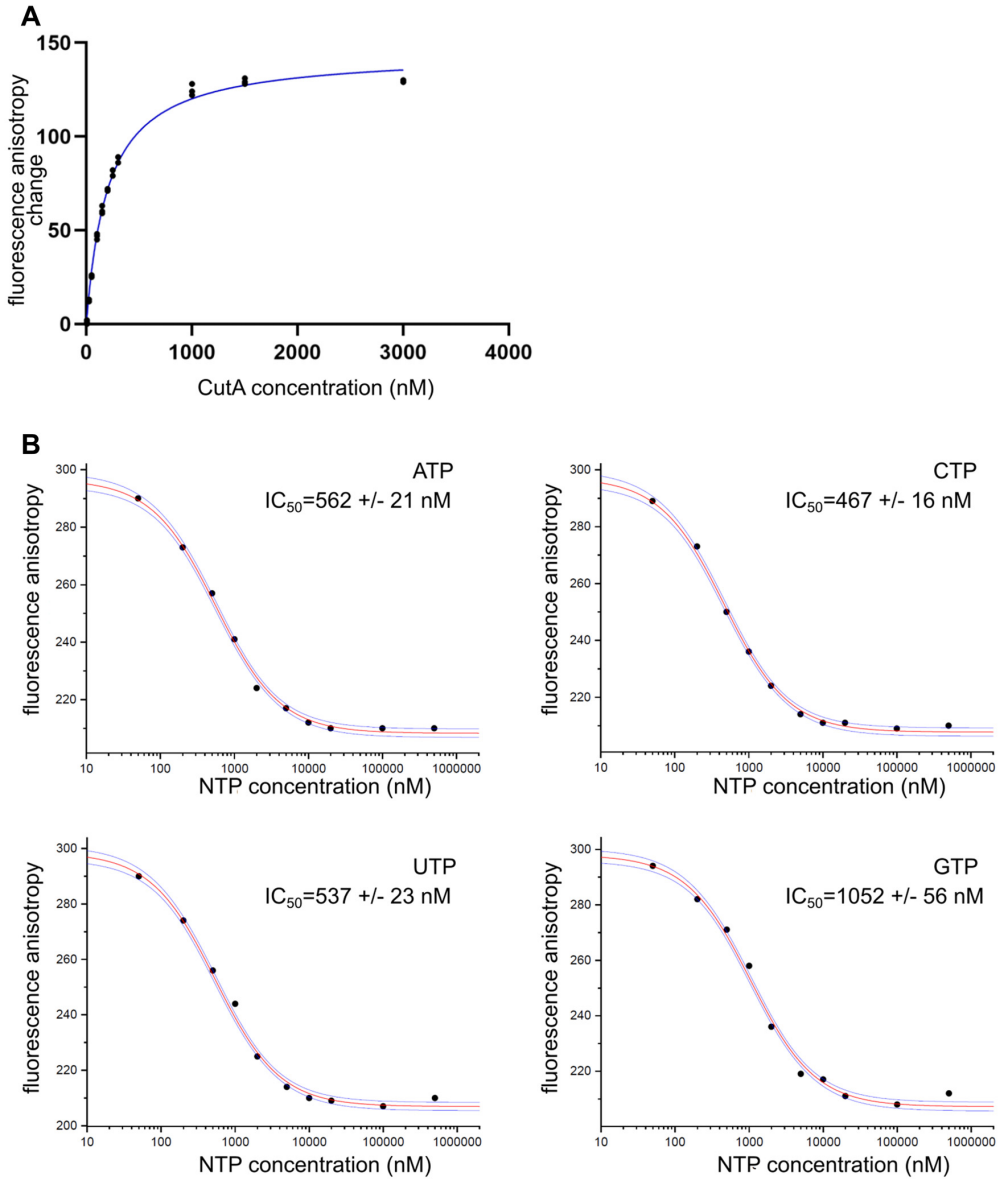
Nucleotide binding by CutA in fluorescence anisotropy experiments. (**A**) Titration of fluorescent ATP analogue with increasing concentration of CutA. Changes in fluorescence anisotropy are plotted against CutA concentration. Three independent measurements are plotted for each concentration. (**B**) Competition assay. CutA was mixed with fluorescent ATP analogue and increasing amounts of NTPs (indicated in each panel) were added. The graphs present data for one repetition (the remaining two are shown in Supplementary Figure S5). The red line is the fit to the data and blue lines represent the confidence bands for the fitted function.

We explored the mechanism that underlies processive adenosine polymerization, the preferential incorporation of cytidine and the lack of activity in the presence of guanosine. One possibility is that CutA interacts with some incoming nucleotides with higher affinity than others. Therefore, we used fluorescence anisotropy measurements to determine the *K*_d_ of a fluorescently modified ATP analog for CutA (Figure 8A). Binding experiments were performed in the presence of Ca^2+^ ions to inhibit any enzymatic activity. The obtained *K*_d_ value was 250 nM. Next, we used a binding competition experimental setup to compare affinity for the four NTPs. A fluorescent ATP analog (50 nM) was mixed with CutA (250 nM), and increasing concentrations of non-fluorescent NTPs were added. Fluorescence anisotropy was measured, and the values were fitted to a competition binding equation. The resulting IC_50_ values were 562 ± 21 nM for ATP, 467 ± 16 nM for CTP, 537 ± 23 nM for UTP and 1052 ± 56 nM for GTP (Figure 8B and Supplementary Figure S5). Therefore, the affinity of CutA for incoming nucleotides was on the order of CTP > ATP = UTP >> GTP.

The lowest affinity for GTP is consistent with our structural data and modeling results, suggesting clashes between the protein and 2-amino group of the guanosine base, which would reduce affinity for this nucleotide. However, this lower affinity does not explain the complete lack of activity for GTP. We hypothesize that when GTP is bound by the protein, its triphosphate group is misaligned at the active site because of these clashes, thus precluding activity. To verify this possibility biochemically, we first tested the incorporation of inosine triphosphate. Inosine has a chemical structure that differs from guanosine only by the absence of the 2-amino group of the base. CutA was able to incorporate inosine into a primer that ended with four adenosines, albeit with relatively low efficiency (Supplementary Figure S6). This showed that removal of the 2-amino group partially rescued enzymatic activity. The 2-NH_2_ group is predicted to form a potential clash with Leu399. We also tested activity of the L399A variant. It showed wild-type levels of adenosine polymerization and, similar to wild-type protein, did not incorporate guanosine phosphate (not shown). Therefore, additional elements of CutA other than the Leu399 side chain may also be involved in selection against the 2-NH_2_ group of the nucleobase.

The aforementioned considerations are consistent with structural data that are available for Cid1. Cid1 binds incoming GTP differently from other NTPs, with the base in syn conformation (25). Such a conformation would alleviate clashes between the protein and 2-NH_2_ group of the base. However, it is unknown whether a syn conformation would also be observed in the presence of the RNA primer and whether CutA uses a similar binding mode for GTP. Remarkably, Cid1 and CutA have different NTP preferences. Cid1 can add one or two guanosines, but it has no activity with CTP (25).

The affinities of CutA for ATP, CTP and UTP were quite similar (Figure 8B). This is consistent with our structures, which did not show any specific contacts with nucleobase of the incoming nucleotide. Slightly higher affinity for CTP may partially explain the preference for its incorporation. However, the processivity of ATP incorporation needs to be explained by factors other than a very high affinity for ATP as the incoming nucleotide. As described above, our structures revealed interactions between the edge of the base of nt −1 and Asn397 and between the base surface and Val377. Both elements are predicted to form stronger interactions with adenine than with pyrimidines (uracil or cytosine). This suggests the higher affinity of CutA for primers that end with adenosines and would explain the processivity of adenosine incorporation. One important element of the mechanism of CutA may be the interplay between the nature of the bound primer and incoming nucleotide. This is consistent with data that showed that the nature of the 3′-terminus of the primer for CutA greatly affects specificity for incorporation of the next nucleotide (34). One can envisage that the adenine base that is bound at position nt -1 and the base of the incoming ATP would form stacking interactions that would be more extensive than if one of the two bases is a smaller pyrimidine. This could lead to the more efficient binding of both ATP and a primer that ends with adenosine, which would be another mechanism of processivity for adenosine polymerization.

Finally, we cannot exclude the possible involvement of more dynamic processes in the preferential incorporation of particular nucleotides. In our crystal structures, we observed alternative conformations of side chains of Arg557 and Glu551 that were located in the vicinity of the base of the incoming nucleotide. A similar dynamic mechanism was proposed for uracil recognition by Cid1, which involved flipping of the imidazole ring of a key histidine residue (His 336, Supplementary Figure S4) (22). Such small conformational changes of the protein or nucleic acid may play a role in sequence discrimination. Careful molecular dynamics simulations need to be performed to decipher these mechanisms.

### Variants of CutA^240-603^ with point substitutions exhibit defects in catalytic activity

To verify the structural data and further study the mechanism of CutA, we performed biochemical assays using variants of CutA with substitutions that were designed based on the solved structures. Three residues were selected for substitutions. The first residue was Asn397, which forms a hydrogen bond with the base of nt −1. It was substituted with alanine, resulting in the N397A variant. The second residue was Ala400, which participates in the formation of a pocket that binds the base of nt −1, the side chain of which is predicted to form a clash with the 2-amino group of guanine, thus selecting against it. We replaced this residue with glycine, resulting in the A400G variant. The third residue was Asn403, which forms hydrogen bonds with the incoming nucleotide (including both the base and 2′-OH group of the ribose). Its replacement with alanine produced the N403A variant. We also included in our analyses two previously characterized variants, R557A and R557H, with substitutions of the residue that were predicted to form base-specific contacts with the incoming nucleotide (34).

**Figure 9.**
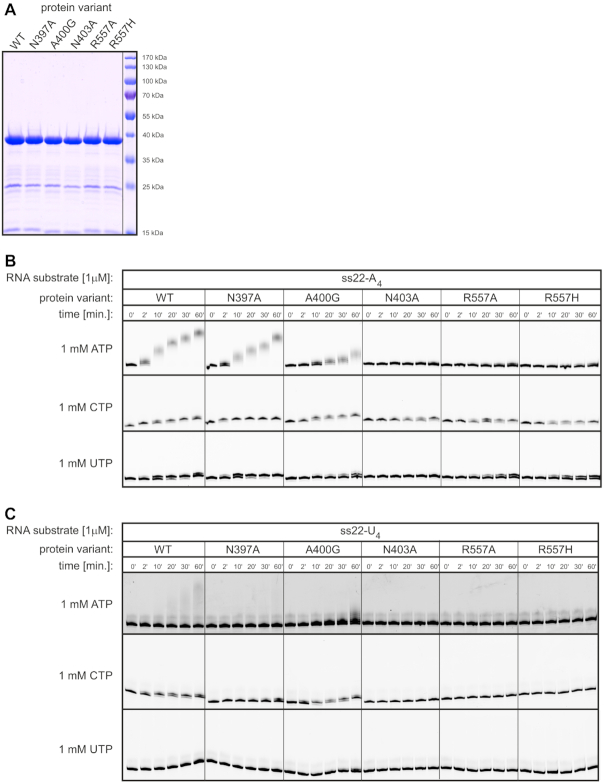
Nucleotidyl transferase assay for wild-type CutA^240–603^ and its variants with point substitutions. (**A**) SDS-PAGE analysis of protein variants that were used in the activity assays. Equal amounts of all proteins were used in the activity assays that are shown in (B) and (C). PageRuler Prestained Protein Ladder (Thermo Fisher Scientific) was run in the rightmost lane. The molecular weight (MW) is indicated on the right. (**B**) Results for the ss22-A_4_ RNA substrate that contained four adenosines at the 3′-end. Enzymatic activity was tested in the presence of 1 μM CutA variants with 1 mM ATP, CTP or UTP as the incoming nucleotide as indicated on the left. Reactions were terminated after the time points that are indicated above the lanes. (**C**) Same conditions as in (B) were used to test CutA activity in the presence of ss22-U_4_ (i.e. RNA primer with four uridines at the 3′-end).

The activity analysis was performed with carefully equalized amounts of CutA variants (Figure 9A). In the first round of experiments, we used an RNA primer that contained four terminal adenosines (Figure 9B). Wild-type protein efficiently added adenosines to this primer (poly[A] polymerase activity). Based on high-resolution gels, ∼11–19 adenosines were added to the RNA in 60 min (Figure 7A). Two cytidines or one uridine were incorporated by the wild-type protein, with the first nucleotide incorporated with 100% efficiency. This is consistent with previous results (34) that showed that CutA is not processive for either UTP or CTP. As described above, the structure offers a potential explanation for this lack of processivity. Once a pyrimidine nucleotide is incorporated into the 3′-terminus, such an RNA primer would have lower affinity for the enzyme and would easily dissociate. This lower affinity would result from less extensive interactions that are formed by smaller pyrimidine bases with the ‘shelf’ element.

The N397A variant retained full activity with ATP, CTP and UTP, with the exception that it was able to add just a single cytidine (Figure 9B). Asn397 is located close to the base of nt −1 and may be involved in sequence specificity, but in this experimental set up defects resulting from its substitution were not apparent. For N403A, the activity with CTP was retained, but the activity with ATP and UTP was completely inhibited. This is consistent with the fact that in our structures and model of a complex with incoming UTP, Asn403 formed hydrogen bonds with the base (N3 of adenine or O2 carbonyl group of uracil) and 2′-OH group of the incoming nucleotide. The A400G variant exhibited lower enzymatic activity for ATP (only a few nucleotides were incorporated) and UTP (1 nt incorporated with ∼40% efficiency after 60 min; Figure 9B). The behavior of the A400G is consistent with the involvement of Ala400 in the formation of the binding pocket for the 3′ terminal adenine of the primer. Interestingly, similar to the N403A variant, the activity of A400G with CTP was less affected. The R557A and R557H variants were completely inactive with ATP. This is in agreement with the structural data. In the CutA-A_3_ structure, Arg557 forms an interaction with the base of the incoming nucleotide participating in its binding. Both Arg557 substitution variants retained partial activity for UTP (one nucleotide incorporated with ∼50% efficiency after 60 min) and full activity for CTP (one nucleotide incorporated with 100% efficiency after 60 min; Figure 9B).

In the second round of experiments, we used a primer that ended with four uridines (Figure 9C). When ATP was used as the incoming nucleotide, wild-type protein incorporated multiple nucleotides but with very low efficiency (Figure 9C). There is apparently a large energetic barrier to start polymerization. However, once the first rounds of the reaction occur, it can proceed in a processive manner. For CTP, 2 nt were incorporated, the first one with 100% efficiency after 60 min. No activity was observed for UTP. The N397A variant was inactive with ATP and UTP but retained some activity for CTP (one nucleotide incorporated with ∼10% efficiency after 60 min; Figure 9C). The other primer-binding pocket variant, A400G, had higher activity with lower processivity for ATP and slightly higher activity for UTP. Activity with CTP was unaffected (Figure 9C). This is consistent with the results that were obtained with the ss22-A_4_ primer. The N403A, R557A and R557H variants were inactive for all three NTPs (Figure 9C). This implies that reactions with a primer that ends with uridine are much more susceptible to defects in NTP binding.

One interesting finding from these experiments is that cytidine incorporation was less affected by substitutions in the protein sequence than the incorporation of adenosine and uridine. This differential effect is difficult to explain based solely on the structures, implying that more subtle effects (e.g. charge distribution in the base, equilibrium between tautomeric forms or dynamic effects) play a role in the binding of incoming nucleotides and the primer. However, the CTP binding which is less sensitive to protein perturbations may explain the preferential incorporation of this nucleotide.

### High-throughput analysis reveals the preference of CutA^240-603^ for cytidine incorporation

Gel-based assays with single NTPs provide only partial insights into the specificity of CutA for nucleotide substrates, particularly given differences in processivity between ATP and CTP or UTP. When the reactions for different CutA variants that were analyzed herein were performed in the presence of the ss22-A_4_ or ss22-U_4_ substrate and either an equimolar AGCU mixture (1:1:1:1) or 10-fold excess of ATP over remaining NTPs (10:1:1:1), changes in processivity compared with the use of single nucleotides were observed (Supplementary Figure S7A and B; compare with Figure 9). For example, the inclusion of equal concentrations of all 4 nt into the reaction that was catalyzed by wild-type CutA and its substitution variants with the ss22-A_4_ primer greatly reduced processivity of the enzyme compared with ATP alone, suggesting that these proteins readily incorporate nucleotides other than adenosine (Supplementary Figure S7A). The R557A and R557H variants, in contrast, produced longer tails in the presence of the AGCU mixture than in the presence of ATP, CTP or UTP alone (Supplementary Figure S7A), clearly indicating that specificity of the analyzed proteins must be more complicated than suggested by the experiments in which single NTP substrates were provided.

To investigate possible changes in specificity for mutant CutA^240–603^ variants relative to wild-type protein, a 3′-RACE-seq experiment was performed. DNA libraries were prepared using reaction products that were obtained for oligoribonucleotide substrates that ended with four adenosines or four uridines in the presence of a mixture of the four NTPs at equal concentrations. A total of approximately 50 000–140 000 reads were included in the analysis, and ∼75% of them were further processed to generate final data for each sample (Supplementary Table S3). The range of mean or median lengths of added 3′-terminal tails that emerged from the analysis of 3′-RACE-seq data was between 0 and 7 nt (Supplementary Figure S8A, *left*). The longest extensions were synthesized by wild-type protein, whereas the N403A variant was nearly completely inactive, regardless of the nature of the RNA primer, which further confirmed the essential role of Asn403 (Supplementary Figure S8A). Qualitatively, the effect of each substitution was similar for ss22-A_4_ and ss22-U_4_. This was expected, based on the CutA–A_3_ structure, because after two nucleotide additions, the initial 3′-terminus of the RNA would move outside the protein interface and no longer influence the reaction. Importantly, the lengths of the tails that were estimated based on the sequencing data corresponded well to extensions that were observed in the gel-based analysis of polymerization products, thus demonstrating that amplification during preparation of the libraries did not introduce much bias (Supplementary Figure S8B).

**Figure 10.**
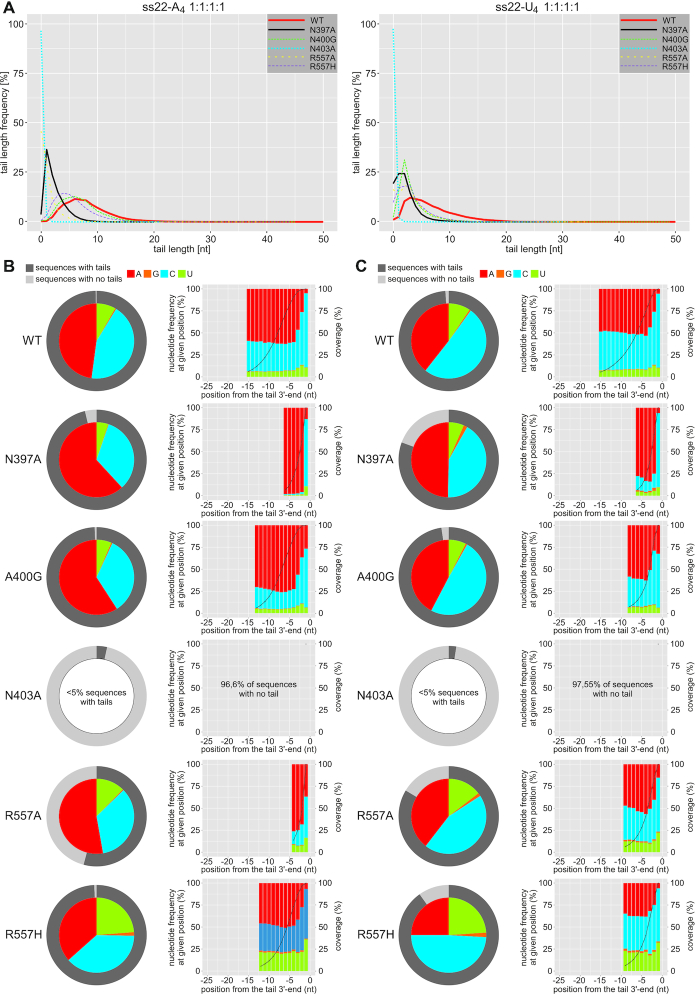
3′-RACE-seq analysis of nucleotide specificity for wild-type CutA^240–603^ and its variants with single amino acid substitutions. (**A**) Length distribution for tails that were added by the indicated CutA^240–603^ variants to the ss22-A_4_ (left) and ss22-U_4_ (right) oligoribonucleotide substrates. (**B** and **C**) Analysis of nucleotide composition for tails that were added by different CutA variants to the ss22-A_4_ (B) and ss22-U_4_ (C) RNA substrates. Outer circles show fractions of sequences that corresponded to tailed and untailed reaction products (dark and light gray, respectively). The inner pie charts show the overall frequency of the incorporation of individual nucleotides into the 3′-terminal extensions. Histograms show the percentage of nucleotide types that were incorporated at the given position of the tail (the last 3′-terminal position is on the right). Only these positions, which were covered by at least 5% of the 3′-RACE-seq reads, are presented in the histograms (the percentage of tails with a nucleotide at every given position is indicated with black dots). The 25 nt limit from the 3′-end of the tail was chosen arbitrarily.

Analysis of the overall frequency of particular nucleotides in the added extensions revealed that, similar to longer CutA variants that were analyzed in our previous study (34), in the presence of equal concentrations of all four NTPs, wild-type CutA^240–603^ protein synthesized tails that contained ∼40–50% cytidines, depending on the substrate (Figure 10). The frequency of guanosine incorporation was <1%, which was consistent with the biochemical data that showed a lack of protein activity toward GTP (34) (Figure 10). The remaining pool of incorporated nucleotides consisted mainly of adenosines and up to ∼9% uridines (Figure 10). Altogether, these results corroborated previous observations that CutA’s specificity for CTP is unusually high among known non-canonical poly(A)/(U) polymerases.

For the ss22-A_4_ primer, N397A substitutions resulted in a modest increase in adenosine incorporation, with a concomitantly lower percentage of cytidines that were present in the synthesized tails (Figure 10). Notably, the tails generated by this variant were shorter confirming the importance of Asn397 for primer binding which was predicted based on the crystal structure of CutA–A_3_ complex, but was not apparent from the gel-based assays with single NTPs (Figure 9B and C). In contrast, A400G substitution exerted a modest effect in the 3′-RACE-seq experiment, with a small increase in adenosine incorporation. For the other primer (ss22-U_4_ RNA), data interpretation was more difficult because of the very low number of reads that corresponded to tailed substrates (<5%).

The R557A and R557H mutations were previously shown to modify the specificity of a longer CutA variant toward adenosine and uridine, respectively (34). Therefore, we included them in the present study as a reference for the newly designed mutants. As expected, the substitution of arginine with alanine increased adenosine incorporation at the expense of cytidine. In turn, the substitution of arginine with histidine led to a pronounced increase in uridine inclusion; in this case, however, the high preference for cytidine was maintained, whereas the percentage of adenosines was substantially reduced (Figure 10). An increase in uridine incorporation is consistent with the fact that histidine in this position has been identified as a determinant of preferential UTP binding in other TENTs (22–26). However, the single R557H substitution did not convert CutA to an enzyme which would incorporate uridines instead of cytosines.

Another important feature of wild-type CutA is a strong preference for cytidine incorporation at the last and penultimate positions of the tail. We recapitulated this finding for the CutA^240–603^ truncation. Importantly, in the present study, this tendency of the mutants appeared to be reduced to a different extent. For example, the N397A variant still incorporated predominantly cytidine at position nt −1, but the preference for cytidine at position nt −2 was much lower than for the wild-type counterpart. The A400G variant incorporated cytidine less frequently as the 3′-terminal nucleotide. For the R557H variant, which incorporated uridines more frequently at every position of the tail compared with wild-type protein, the highest uridine incorporation was observed for the last position.

In summary, our results showed that single amino acid substitutions were insufficient to radically change the properties of the polymerase activity of CutA. Similar conclusions were previously drawn based on CutA variants with multiple substitutions (34). This further strengthens the hypothesis that combinations of factors and not merely protein sequence in key positions determine the biochemical properties of CutA and, more generally, terminal ribonucleotide transferases.

## CONCLUSION

The present study extensively characterized CutA TENT both structurally and biochemically. To our knowledge, this is the first structural characterization of a TENT adding C/U tails. We sought to elucidate the structural basis of several key unique features of CutA, including processive RNA polymerization exclusively for adenines, a complete lack of activity for GTP as the incoming nucleotide, an inability to extend primers that end with guanosines, and a preference for the incorporation of cytidines over uridines. This last feature is interesting because this is a unique property of CutA. The two structures we present herein reveal snapshots of the binding of adenine and cytosine as nucleobases of the incoming nucleotide and binding of the poly(A) primer. Based on the structures and simple modeling exercises, in which we replaced bases of the incoming nucleotide or in the 3′-terminal nucleotide of the primer, we identified elements that likely participate in base recognition. The binding of GTP or a primer with a terminal guanosine is predicted to lead to clashes between NH_2_ of the guanine and the protein, which may explain why CutA is unable to use these ligands as substrates. Our structural and biochemical findings imply that the biochemical properties of CutA result from complex interactions among several factors. For example, we hypothesize that processive adenosine incorporation likely results from tighter binding of the primer with 3′-terminal adenosine and efficient stacking between adenosine bases of the primer and incoming ATP. Nonetheless, the structural, modeling and biochemical data do not provide a clear explanation for the preference of CutA for the incorporation of cytosines and uracils. For example, we found that CTP and UTP were bound by CutA with similar affinities. Thus, factors other than simple differences in NTP affinity must be involved in this preference. The diversity of side chain conformations of the NTP-binding pocket that was observed in our structures implies a more dynamic process of NTP recognition. Further studies will provide additional insights into this unique recognition mechanism.

## DATA AVAILABILITY

Atomic coordinates and structure factors for the reported crystal structures have been deposited in PDB (IDs: 6YWP [apo CutA], 6YWN [CutA–CMPCPP complex] and 6YWO [CutA–A_3_ RNA complex]).

## Supplementary Material

gkaa647_Supplemental_File
