# Supplementary material for: Structure and mechanism of CutA, RNA nucleotidyl transferase with an unusual preference for cytosine
Source: Nucleic Acids Res. 2020 Aug 12;48(16):9387–405. doi: 10.1093/nar/gkaa647 (PMC7498324; doi:10.1093/nar/gkaa647)
Supplement: gkaa647_Supplemental_File [file gkaa647_supplemental_file.pdf]

Structure and mechanism of CutA, RNA nucleotidyl transferase with an unusual preference for cytosine

Deepshikha Malik, Kamil Kobyłecki, Paweł Krawczyk, Jarosław Poznański, Aleksandra Jakielaszek, Agnieszka Napiórkowska, Andrzej Dziembowski, Rafał Tomecki and Marcin Nowotny

SUPPLEMENTARY DATA

Supplementary Tables...2

Supplementary Figures...6

Supplementary References..16

Supplementary Tables

SUPPLEMENTARY TABLE S1. Plasmids used in this study.

**Plasmid**	**Source or genotype**
pET28M N-6xHis-SUMOTag	(Lebreton et al, 2008; Malakhov et al, 2004)
pCC19	[pET28M N-6xHis-SUMOTag] TtCutA$^{276-593}$
pCC20	[pET28M N-6xHis-SUMOTag] TtCutA$^{235-605}$
pCC21	[pET28M N-6xHis-SUMOTag] TtCutA$^{133-765}$
pCC22	[pET28M N-6xHis-SUMOTag] TtCutA$^{274-585}$
pCC23	[pET28M N-6xHis-SUMOTag] TtCutA$^{279-583}$
pCC24	[pET28M N-6xHis-SUMOTag] TtCutA$^{322-590}$
pCC25	[pET28M N-6xHis-SUMOTag] TtCutA$^{240-603}$
pCC26	[pET28M N-6xHis-SUMOTag] TtCutA$^{272-599}$
pCC27	[pET28M N-6xHis-SUMOTag] TtCutA$^{272-724}$
pCC28	[pET28M N-6xHis-SUMOTag] TtCutA$^{269-721}$
pCC29	[pET28M N-6xHis-SUMOTag] TtCutA$^{269-596}$
pCC30	[pET28M N-6xHis-SUMOTag] TtCutA$^{273-582}$
pCC31	[pET28M N-6xHis-SUMOTag] TtCutA$^{278-600}$
pCC32	[pET28M N-6xHis-SUMOTag] TtCutA$^{270-596}$
pCC33	[pET28M N-6xHis-SUMOTag] TtCutA$^{240-603}$ N397A
pCC34	[pET28M N-6xHis-SUMOTag] TtCutA$^{240-603}$ A400G
pCC35	[pET28M N-6xHis-SUMOTag] TtCutA$^{240-603}$ N403A
pCC36	[pET28M N-6xHis-SUMOTag] TtCutA$^{240-603}$ R557A
pCC37	[pET28M N-6xHis-SUMOTag] TtCutA$^{240-603}$ R557H

SUPPLEMENTARY TABLE S2. Oligonucleotides used in this study.

Oligonucleotide	Sequence (5'-3')	Purpose
Oligonucleotides for cloning and site-directed mutagenesis		
280_1Sf	gaagtctaccaggaacaaaccggt**ggatcc**GTTGAACGTAAACTGGAAAC	cloning of TtCutA$^{276\text{-}593}$ into pET28M N-6xHis-SUMOTag
280_1Sr	tcagtggtggtggtggtggtg**ctcgag***tta*CTGTTCACAGCATTCTTCCAG	cloning of TtCutA$^{276\text{-}593}$ into pET28M N-6xHis-SUMOTag
280_2Sf	gaagtctaccaggaacaaaccggt**ggatcc**CTGGGTGGCTGTCGCTTCG	cloning of TtCutA$^{235\text{-}605}$ into pET28M N-6xHis-SUMOTag
280_2Sr	tcagtggtggtggtggtggtg**ctcgag***tta*CTGCCAAACGCGTTCTTC	cloning of TtCutA$^{235\text{-}605}$ into pET28M N-6xHis-SUMOTag
280_3Sf	gaagtctaccaggaacaaaccggt**ggatcc**GAACCGGCAGAACGCGCTC	cloning of TtCutA$^{133\text{-}765}$ into pET28M N-6xHis-SUMOTag
280_3Sr	tcagtggtggtggtggtggtg**ctcgag***tta*ATGCTGGATCGGGAAGCC	cloning of TtCutA$^{133\text{-}765}$ into pET28M N-6xHis-SUMOTag
280_4Sf	gaagtctaccaggaacaaaccggt**ggatcc**GAAGACGTTGAACGTAAAC	cloning of TtCutA$^{274\text{-}585}$ into pET28M N-6xHis-SUMOTag
280_4Sr	tcagtggtggtggtggtggtg**ctcgag***tta*GCCTTCAGCAATCAGATC	cloning of TtCutA$^{274\text{-}585}$ into pET28M N-6xHis-SUMOTag
280_5Sf	gaagtctaccaggaacaaaccggt**ggatcc**AAACTGGAAACGGATATG	cloning of TtCutA$^{279\text{-}583}$ into pET28M N-6xHis-SUMOTag
280_5Sr	tcagtggtggtggtggtggtg**ctcgag***tta*AGCAATCAGATCAAATGC	cloning of TtCutA$^{279\text{-}583}$ into pET28M N-6xHis-SUMOTag
280_6Sf	gaagtctaccaggaacaaaccggt**ggatcc**CGTGTTCACCTGTTTGG	cloning of TtCutA$^{322\text{-}590}$ into pET28M N-6xHis-SUMOTag
280_6Sr	tcagtggtggtggtggtggtg**ctcgag***tta*GCATTCTTCCAGTTTGCC	cloning of TtCutA$^{322\text{-}590}$ into pET28M N-6xHis-SUMOTag
280_7Sf	gaagtctaccaggaacaaaccggt**ggatcc**TTCGAAACGGCAATTCCG	cloning of TtCutA$^{240\text{-}603}$ into pET28M N-6xHis-SUMOTag
280_7Sr	tcagtggtggtggtggtggtg**ctcgag***tta*AACGCGTTCTTCTTCTTTC	cloning of TtCutA$^{240\text{-}603}$ into pET28M N-6xHis-SUMOTag
280_8Sf	gaagtctaccaggaacaaaccggt**ggatcc**CTGTCCGAAGACGTTGAACG	cloning of TtCutA$^{272\text{-}599}$ and TtCutA$^{272\text{-}724}$ into pET28M N-6xHis-SUMOTag
280_8Sr	tcagtggtggtggtggtggtg**ctcgag***tta*TTCTTTCGGAAACACATAC	cloning of TtCutA$^{272\text{-}599}$ into pET28M N-6xHis-SUMOTag
280_9Sr	tcagtggtggtggtggtggtg**ctcgag***tta*AGCGTGTGCCAGAACCTGTTGC	cloning of TtCutA$^{272\text{-}724}$ into pET28M N-6xHis-SUMOTag
280_10Sf	gaagtctaccaggaacaaaccggt**ggatcc**AAGAGTAAACTGTCCGAAG	cloning of TtCutA$^{269\text{-}721}$ and TtCutA$^{269\text{-}596}$ into pET28M N-6xHis-SUMOTag
280_10Sr	tcagtggtggtggtggtggtg**ctcgag***tta*CAGAACCTGTTGCTGATTC	cloning of TtCutA$^{269\text{-}721}$ into pET28M N-6xHis-SUMOTag
280_11Sr	tcagtggtggtggtggtggtg**ctcgag***tta*AAACACATACTGTTCACAGC	cloning of TtCutA$^{269\text{-}596}$ and TtCutA$^{270\text{-}596}$ into pET28M N-6xHis-SUMOTag
280_12Sf	gaagtctaccaggaacaaaccggt**ggatcc**TCCGAAGACGTTGAACGTAAAC	cloning of TtCutA$^{273\text{-}582}$ into pET28M N-6xHis-SUMOTag
280_12Sr	tcagtggtggtggtggtggtg**ctcgag***tta*AATCAGATCAAATGCGCG	cloning of TtCutA$^{273\text{-}582}$ into pET28M N-6xHis-SUMOTag
280_13Sf	gaagtctaccaggaacaaaccggt**ggatcc**CGTAAACTGGAAACGGATATG	cloning of TtCutA$^{278\text{-}600}$ into pET28M N-6xHis-SUMOTag
280_13Sr	tcagtggtggtggtggtggtg**ctcgag***tta*TTCTTCTTTCGGAAACAC	cloning of TtCutA$^{278\text{-}600}$ into pET28M N-6xHis-SUMOTag
280_14Sf	gaagtctaccaggaacaaaccggt**ggatcc**AGTAAACTGTCCGAAGACG	cloning of TtCutA$^{270\text{-}596}$ into pET28M N-6xHis-SUMOTag

mutN397Af	catgcgacatgaacgttaacgctaccctggctctggaaaata	site-directed mutagenesis N397A
mutN397Ar	tattttccagagccagggtagcgttaacgttcatgtcgcatg	site-directed mutagenesis N397A
mutA400Gf	cgttaacaataccctgggtctggaaaatacccgca	site-directed mutagenesis A400G
mutA400Gr	tgcgggtattttccagacccagggtattgttaacg	site-directed mutagenesis A400G
mutN403Af	acaataccctggctctggaagctacccgcatggtcc	site-directed mutagenesis N403A
mutN403Ar	ggaccatgcgggtagcttccagagccagggtattgt	site-directed mutagenesis N403A
mutR557Af	gaaccgtttaatgatggtgccaacctgggcaataccg	site-directed mutagenesis R557A
mutR557Ar	cggtattgcccaggttggcaccatcattaaacggttc	site-directed mutagenesis R557A
mutR557Hf	accgtttaatgatggtcacaacctgggcaataccg	site-directed mutagenesis R557H
mutR557Hr	cggtattgcccaggttgtgaccatcattaaacggt	site-directed mutagenesis R557H
Sequencing primers		
SumoF	TCATACTGTCAAAGACAGGG	Sequencing of inserts in pET28M N-6xHis-SUMOTag
T7terminator	TGCTAGTTATTGCTCAGCGG	Sequencing of inserts in pET28M N-6xHis-SUMOTag
280seq5	CAGCATTCTTCCAGTTTGCC	Sequencing of TtCutA
12_109_s1	GCAGCGCCTATTCTGAAACC	Sequencing of TtCutA
12_109_s2	TATCAAATACTGGACCCGCC	Sequencing of TtCutA
12_109_s3	CCGCGGTAACCGTCAGTTTC	Sequencing of TtCutA
RNA oligonucleotides		
5'-FAM-ss22-A_4	FAM-r(**CGACUGGAGCACGAGGACACUG**AAAA)	RNA substrate used in biochemical assays
5'-FAM-ss22-U_4	FAM-r(**CGACUGGAGCACGAGGACACUG**UUUU)	RNA substrate used in biochemical assays
5'-FAM-ss22-C_4	FAM-r(**CGACUGGAGCACGAGGACACUG**CCCC)	RNA substrate used in biochemical assays
5'-FAM-ss22-G_4	FAM-r(**CGACUGGAGCACGAGGACACUG**GGGG)	RNA substrate used in biochemical assays
5'-FAM-ss22	FAM-r(**CGACUGGAGCACGAGGACACUG**)	RNA substrate used in biochemical assays
5'-FAM-ss22-A_1	FAM-r(**CGACUGGAGCACGAGGACACUG**A)	RNA substrate used in biochemical assays
5'-FAM-ss22-A_2	FAM-r(**CGACUGGAGCACGAGGACACUG**AA)	RNA substrate used in biochemical assays
5'-FAM-ss22-A_{10}	FAM-r(**CGACUGGAGCACGAGGACACUG**AAAAAAAAAA)	RNA substrate used in biochemical assays
Oligonucleotides used in the 3'-RACE-seq procedure		
RA3	TGGAATTCTCGGGTGCCAAGG	Adapter oligonucleotide
RTP	*GCCTTGGCACCCGAGAATTCCA*	Reverse transcription primer
RA5_ss22	*GTTCAGAGTTCTACAGTCCGA*CGATC**CGACTGGAGCACGAGGACACTG**	First-round PCR primer (forward)
RP1	AATGATACGGCGACCACCGAGATCTACAC*GTTCAGAGTTCTACAGTCCGA*	Second-round PCR primer (forward)
RPI_X	CAAGCAGAAGACGGCATACGAGATNNNNNNGTGACTGGAGTT*CCTTGGCACCCGAGAATTCCA*	PCR primer with RNA-seq index (reverse)

SUPPLEMENTARY TABLE S3. General information on the 3'-RACE-seq data obtained for products of reactions catalyzed by various truncated CutA variants with point mutations in the presence of equimolar NTP concentration.

sample	no of raw reads	no of processed reads	percentage of analyzed reads
WT ss22-A4	97067	72287	74,47
N397A ss22-A4	85164	65352	76,74
A400G ss22-A4	104222	76929	73,81
N403A ss22-A4	101276	77101	76,13
R557A ss22-A4	141054	108851	77,17
R557H ss22-A4	122008	91159	74,72
WT ss22-U4	86672	66222	76,41
N397A ss22-U4	53575	40865	76,28
A400G ss22-U4	82503	62932	76,28
N403A ss22-U4	78736	59804	75,96
R557A ss22-U4	65513	50746	77,46
R557H ss22-U4	51506	39189	76,09

Supplementary Figures

A

name	N-terminus	C-terminus	secondary structure prediction based on:	remarks
TtCutA FL	1	1275		full-length protein
TtCutA_tr1	216	617		variant utilized previously
TtCutA_tr3	127	827		variant utilized previously, *i.a.* for introduction of amino acid substitutions
TtCutA 276-593	276	593		entire PAP/OAS1 substrate-binding domain (SBD) with inserted NTase domain
TtCutA 235-605	235	605	CFSSP: http://www.biogem.org/tool/chou-fasman/	similar to TtCutA_tr1, truncated to boundaries of predicted secondary structures
TtCutA 133-765	133	765	CFSSP: http://www.biogem.org/tool/chou-fasman/	similar to TtCutA_tr3, truncated to boundaries of predicted secondary structures
TtCutA 274-585	274	585	SWISS-MODEL: https://swissmodel.expasy.org/interactive	according to model based on *S. pombe* Cid1; more probable secondary structure boundaries
TtCutA 279-583	279	583	SWISS-MODEL: https://swissmodel.expasy.org/interactive	according to model based on *S. pombe* Cid1; less probable secondary structure boundaries
TtCutA 322-590	322	590	SWISS-MODEL: https://swissmodel.expasy.org/interactive	according to model based on *C. elegans* Gld-2
			CPHmodels: http://www.cbs.dtu.dk/services/CPHmodels/	
TtCutA 240-603	240	603	GlobPlot: http://globplot.embl.de/	
TtCutA 272-599	272	599	RaptorX: http://raptorx.uchicago.edu/StructurePrediction/predict/	one predicted structural unit
TtCutA 272-724	272	724	RaptorX: http://raptorx.uchicago.edu/StructurePrediction/predict/	two neighbouring predicted structural units (272-599 and 669-724)
TtCutA 269-721	269	721	Phyre 2http://www.sbg.bio.ic.ac.uk/~phyre2/html/page.cgi?id=index	longer less probable variant
TtCutA 269-596	269	596	Phyre2: http://www.sbg.bio.ic.ac.uk/~phyre2/html/page.cgi?id=index	shorter less probable variant
TtCutA 273-582	273	582	Phyre2: http://www.sbg.bio.ic.ac.uk/~phyre2/html/page.cgi?id=index	most probable variant
TtCutA 278-600	278	600	HHPRED: https://toolkit.tuebingen.mpg.de/tools/hhpred	
TtCutA 270-596	270	596	LOMETS: https://zhanglab.ccmb.med.umich.edu/LOMETS/	

B

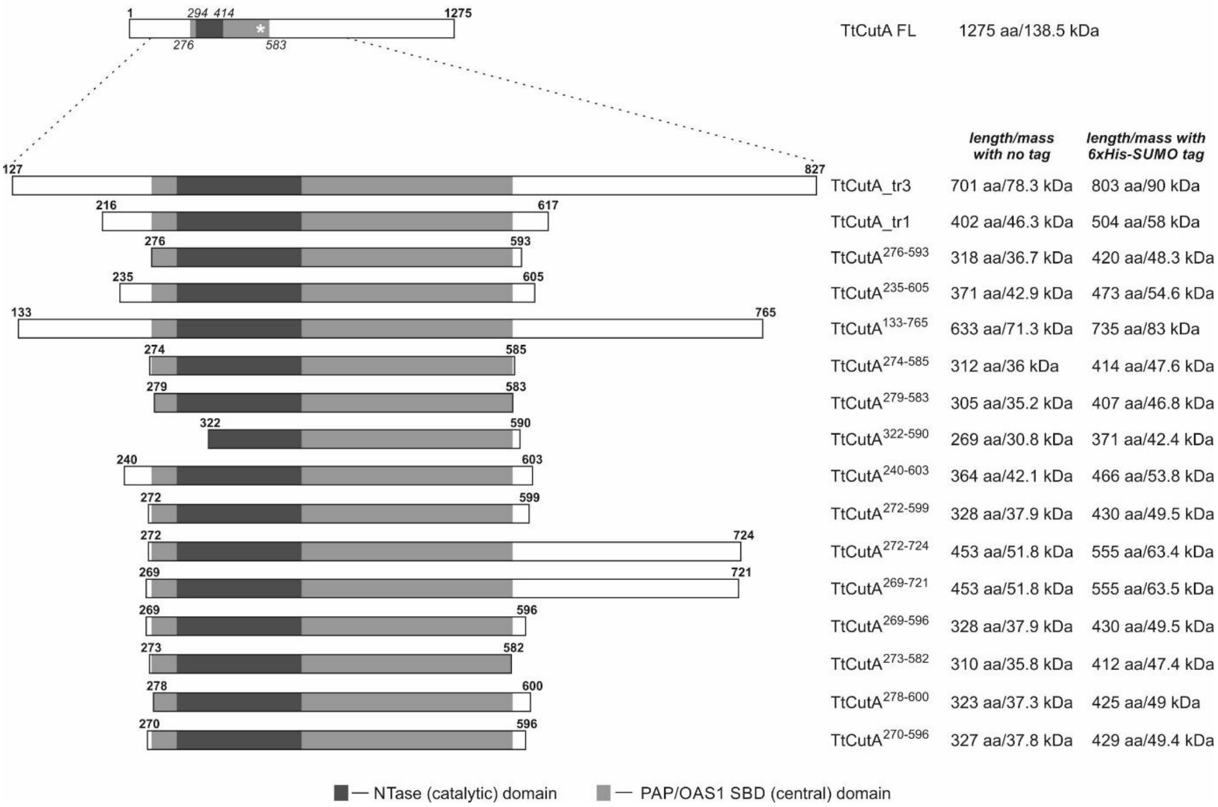

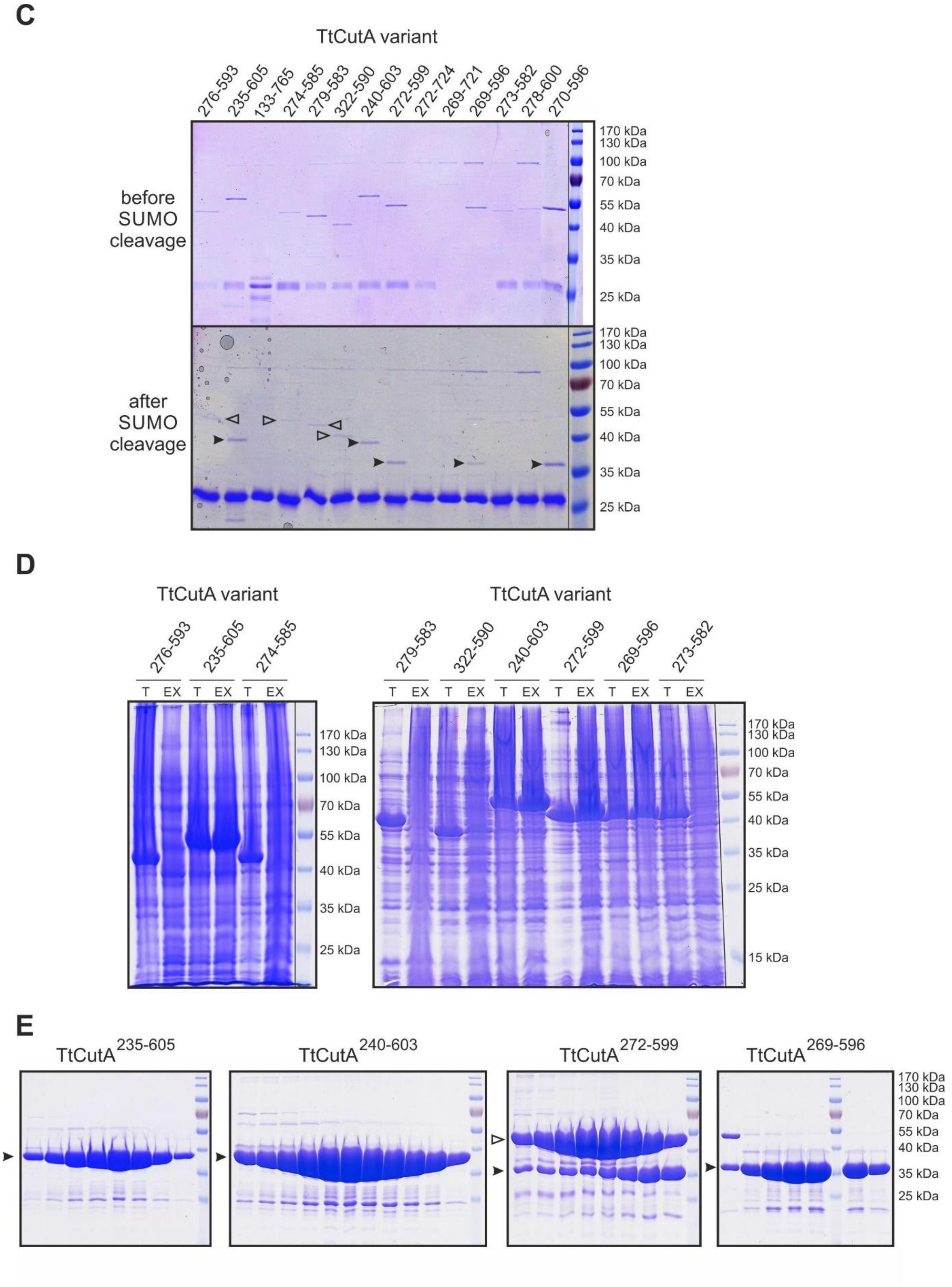

Supplementary Figure S1 (*previous two pages*). An overview of methodology for design and purification of truncated *T. terrestris* CutA (TtCutA) that could be used in crystallization trials. (**A**) A list of different TtCutA variants, including full-length protein, previously used truncations and 14 novel shortened versions. Positions of N and C boundaries, names of publicly available programs for prediction of protein secondary structures along with respective URLs, and additional remarks are indicated. (**B**) A scheme of full-length TtCutA (*top*) and truncated protein variants (*bottom*; zoomed).

Nucleotidyl transferase (NTase) and inserted PAP/OAS1 SBD domains (*i.e.* palm and fingers domains, respectively) are indicated with light and dark grey, respectively. Position of the nucleotide recognition motif (NRM) is marked with an asterisk in the TtCutA FL. Lengths and molecular masses of the protein variants with no tag and with N-terminal 6xHis-SUMO tag are shown in the right. (**C**) Results of small-scale purification of 14 truncated TtCutA variants performed using Protino 96 Ni-IDA plate (SDS-PAGE analysis of protein samples). Upper part shows eluates from nickel column prior to SUMO protease cleavage. Variants 133-765, 272-724 and 269-721, with C-terminus significantly extended downstream NTase domain, were hardly visible. Lower part demonstrates eluted proteins after SUMO protease cleavage. Positions of bands corresponding to uncleaved and cleaved proteins are marked with open and closed arrowheads, respectively. Only 235-605, 240-603, 272-599, 269-596 and 270-596 variants were cleaved efficiently. (**D**) Analysis of protein solubility following large-scale expression. Total protein extracts and supernatants after cell disruption and ultracentrifugation from bacteria producing selected TtCutA variants were analyzed alongside. 235-605, 240-603, 272-599 and 269-596 variants proved to be soluble. (**E**) 235-605, 240-603, 272-599 and 269-596 variants were purified by nickel affinity chromatography followed by size exclusion. Fractions after gel filtration were analyzed by SDS-PAGE. 272-599 variant was not efficiently cleaved. Out of remaining three variants, 240-603 truncation yielded highest amounts of a relatively pure and stable protein. 235-605, 240-603 and 269-596 variants were subjected to crystallization trials, which resulted in promising crystals only for 240-603 truncation.

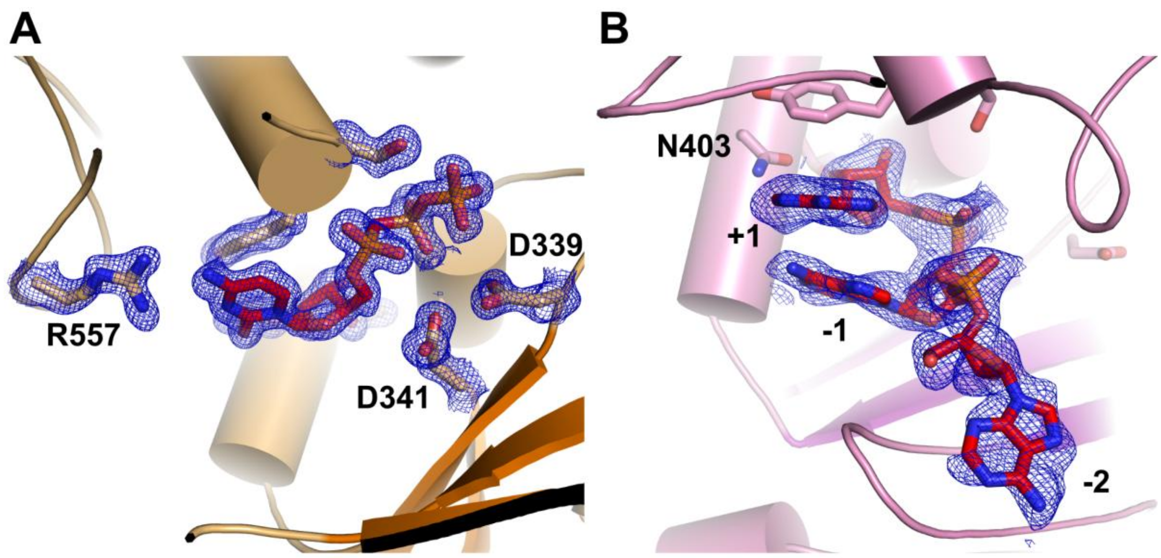

Supplementary Figure S2. Samples of 2Fo-Fc simulated annealing composite omit electron density maps. (**A**) Close-up of the active site of the CutA-CMPCPP structures. The map is contoured at 1.2 σ. (**B**) RNA bound in the CutA-A_3 structure. Map contoured at 0.8 σ.

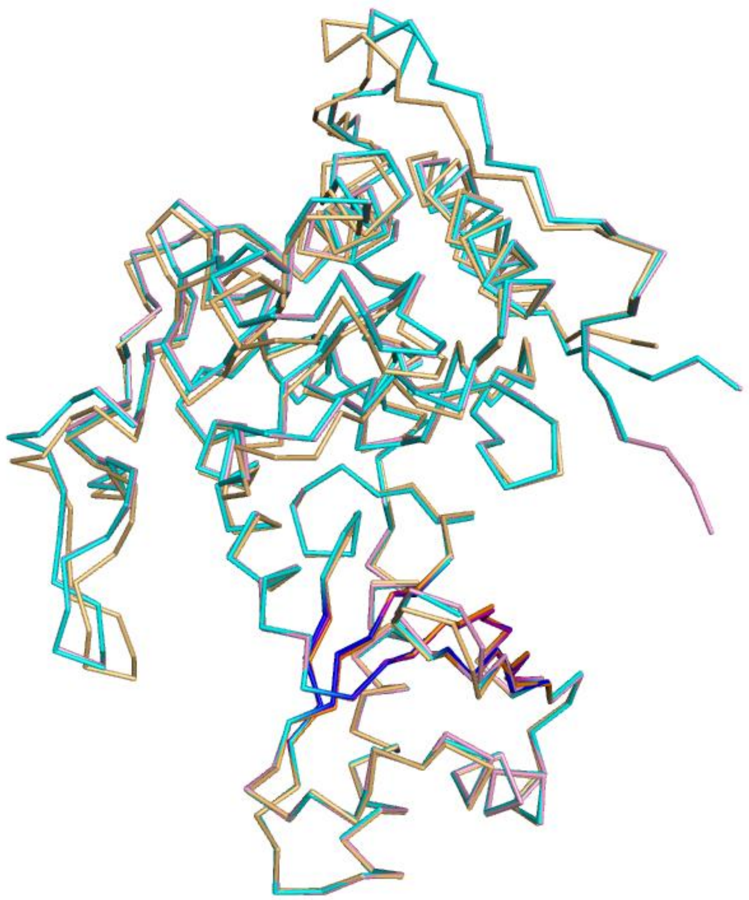

Supplementary Figure S3. Comparison of the CutA conformation in apo form and in complex with CMPCPP and A_3 RNA. The protein chains were superimposed based on the palm subdomain and are shown in wire representation. Protein subunit from the structure of CutA-CMPCPP complex is in orange with β-strands in darker shade of orange. Protein subunit from CutA-A_3 complex is in pink with β-strands in purple. Protein from apo structure is in cyan with β-strands in blue.

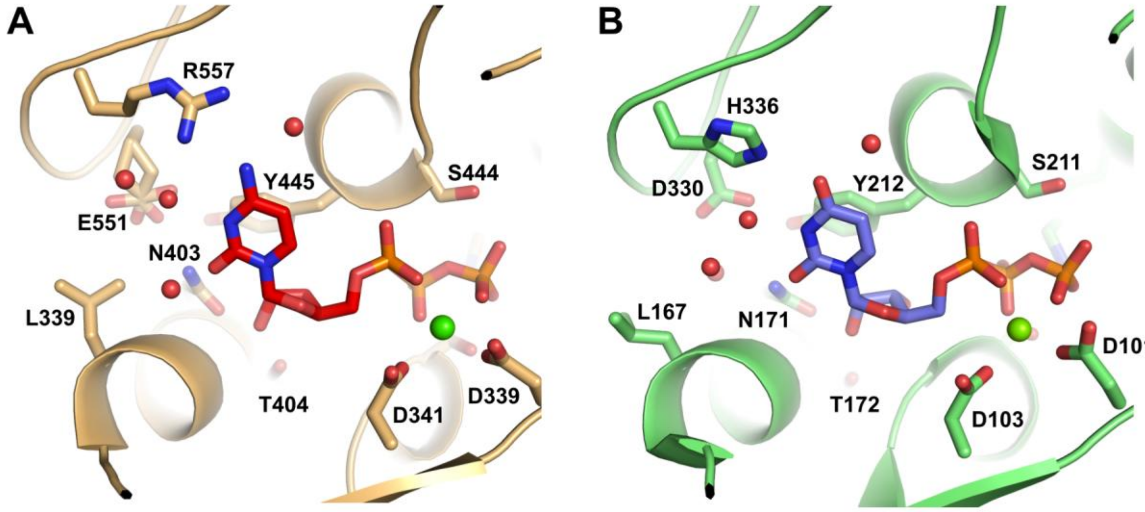

Supplementary Figure S4. Comparison of the active sites of CutA (**A**; complex with CMPCPP) and Cid1 (**B**; complex with UTP, PDB ID: 4FH5). The proteins are shown in cartoon representation, selected active site residues are shown as sticks. Metal ions (Ca^{2+} for CutA and Mg^{2+} for Cid1) are shown as green spheres. Selected water molecules that interact with the nucleotide are shown as red spheres.

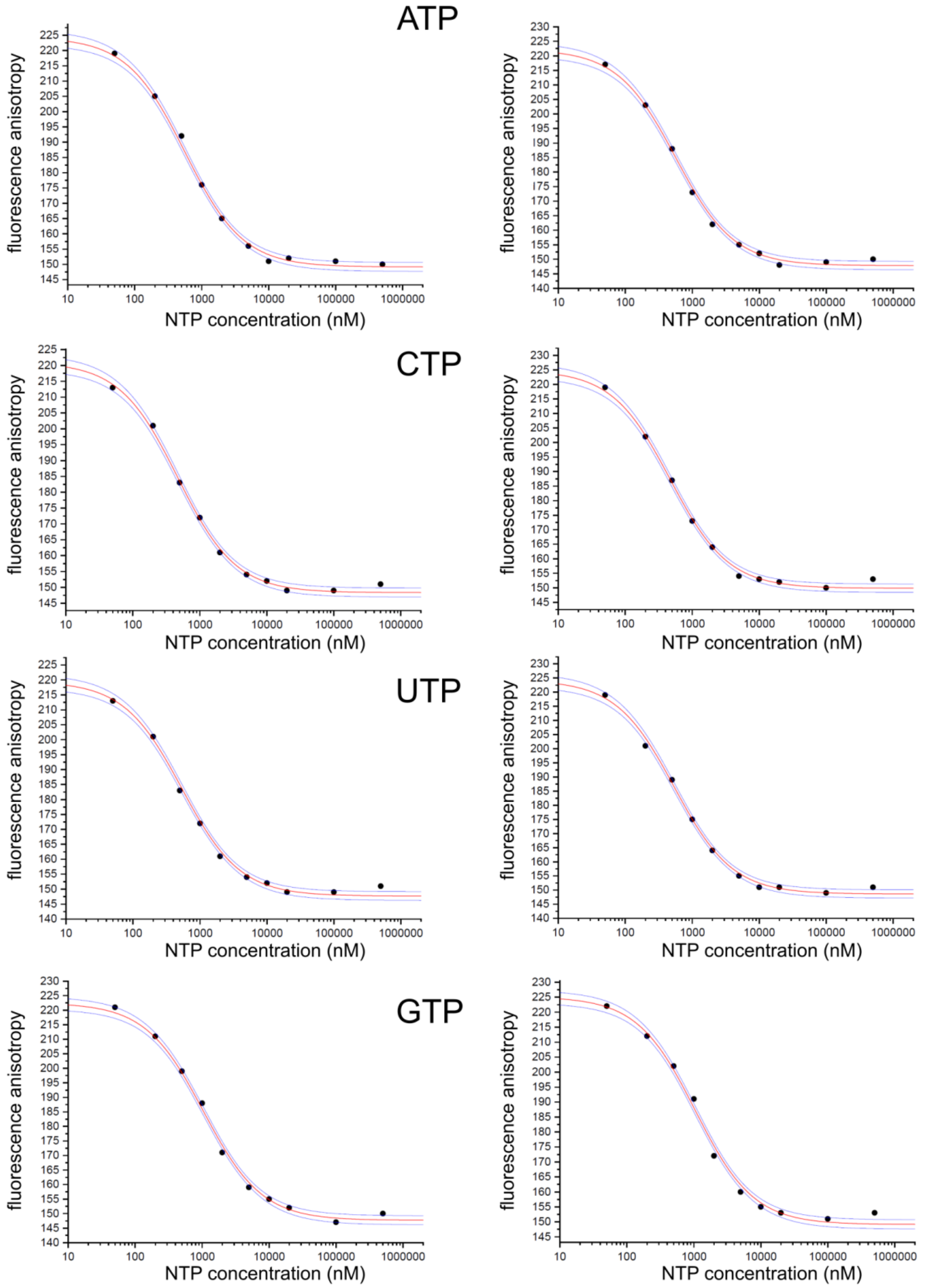

Supplementary Figure S5. Additional repetitions of the NTP competition experiments (see legend of Fig 8).

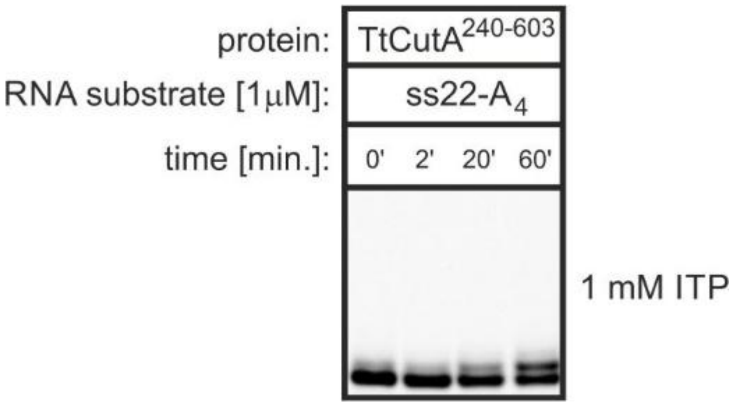

Supplementary Figure S6. Analysis of ITP incorporation by TtCutA$^{240\text{-}603}$ protein variant. Enzymatic activity assay was performed for ss22-A_4 RNA primer in the presence of 1 μM truncated CutA variant with 1 mM ITP as the incoming nucleotide. Reaction was terminated after the time points indicated above the lanes.

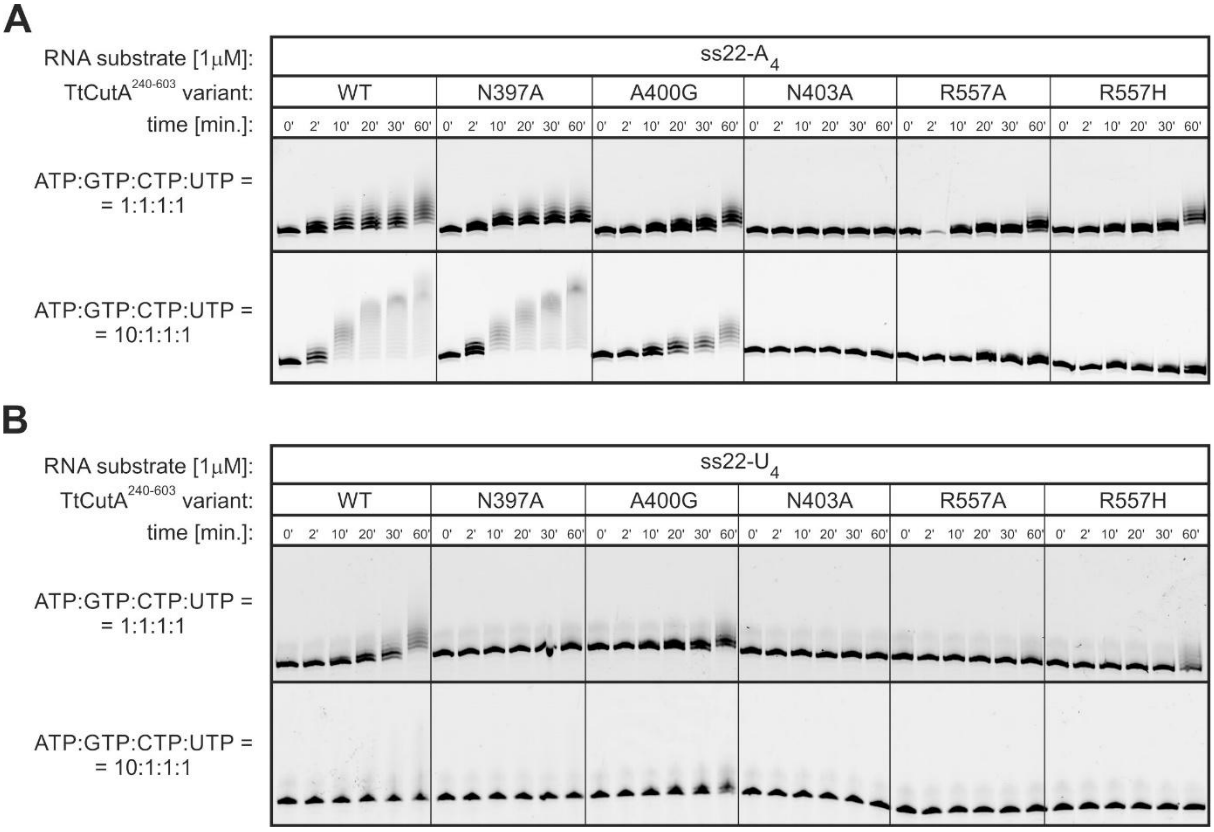

Supplementary Figure S7. Enzymatic activity of TtCutA$^{240\text{-}603}$ wild-type variant and its counterparts with point substitutions in the presence of NTP mixtures. (**A**) Results for ss22-A_4 RNA substrate, containing four adenosines at the 3'-end. The enzymatic activity was tested in presence of 1 μM CutA variants with an equimolar NTP mixture (*top*) or with 10-fold excess of ATP over other nucleotides (*bottom*), as indicated on the left. Reactions were terminated after the time points indicated above the lanes. (**B**) Same conditions as in (A) were used for testing CutA activity in presence of ss22-U_4, *i.e.* RNA primer with four uridines at 3' end.

A

sample	mean tail length	median tail length
WT ss22-A4	7,99	7
N397A ss22-A4	2,37	2
A400G ss22-A4	6,84	6
N403A ss22-A4	0,04	0
R557A ss22-A4	1,1	1
R557H ss22-A4	5,64	5
WT ss22-U4	6,61	6
N397A ss22-U4	2,1	2
A400G ss22-U4	3,31	3
N403A ss22-U4	0,04	0
R557A ss22-U4	3,13	3
R557H ss22-U4	3,34	3

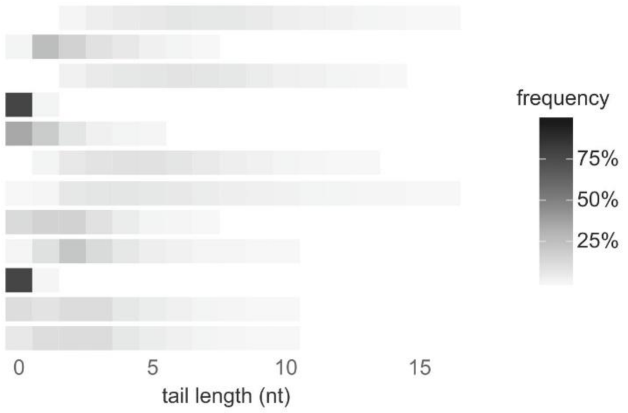

B

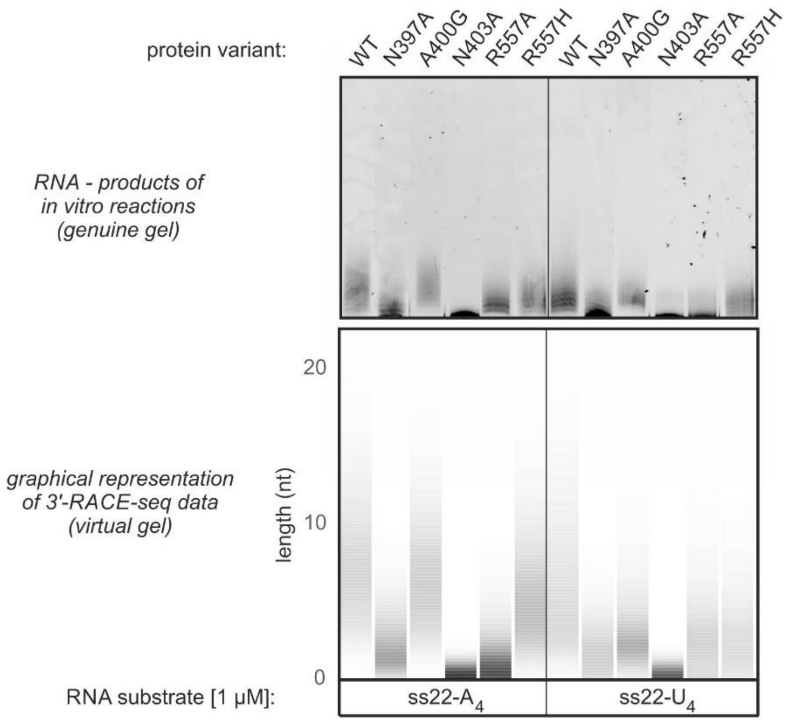

Supplementary Figure S8. Lengths of tails added by WT CutA and its counterparts with point substitutions inferred based on 3'-RACE-seq data analysis. (**A**) Table on the left contains information on mean and median tail lengths for each sample. Heat map on the right demonstrates frequency of tails containing a given number of nucleotides added by respective protein variants. (**B**) Comparison of PAGE gel with genuine reaction products utilized for 3'-RACE-seq library preparation (*top*) and virtual gel representing data from high-throughput sequencing (*bottom*).

Supplementary References

1. Lebreton,A., Tomecki,R., Dziembowski,A. and Séraphin,B. (2008) Endonucleolytic RNA cleavage by a eukaryotic exosome. *Nature*, **456**, 993–996.

2. Malakhov,M.P., Mattern,M.R., Malakhova,O.A., Drinker,M., Weeks,S.D. and Butt,T.R. (2004) SUMO fusions and SUMO-specific protease for efficient expression and purification of proteins. *J. Struct. Funct. Genomics*, **5**, 75–86.
